# Preoperative Ultrasound for the Prediction of Postinduction Hypotension: A Systematic Review and Meta-Analysis

**DOI:** 10.3390/jpm14050452

**Published:** 2024-04-25

**Authors:** Chunyu Liu, Ran An, Hongliang Liu

**Affiliations:** Department of Anesthesiology, Chongqing University Cancer Hospital, Chongqing 400030, China; liucy@cqu.edu.cn (C.L.); anran1011@cqu.edu.cn (R.A.)

**Keywords:** postinduction hypotension, ultrasound, meta-analysis

## Abstract

Postinduction hypotension (PIH) is closely associated with postoperative adverse outcomes. Preoperative hypovolemia is a key risk factor, and many parameters are available from ultrasound to detect hypovolemia, but the accuracy of PIH from ultrasound remains unclear. This systematic review and meta-analysis aimed to evaluate the commonly used measurements from ultrasound to predict PIH. We searched the PubMed, Cochrane Library, Embase, CNKI, and Web of Science databases from their inception to December 2023. Thirty-six studies were included for quantitative analysis. The pooled sensitivities for the inferior vena cava collapsibility index (IVC-CI), maximum inferior vena cava diameter (DIVCmax), minimum inferior vena cava diameter (DIVCmin), and carotid artery corrected flow time (FTc) were 0.73 (95% CI = 0.65, 0.79), 0.66 (95% CI = 0.54, 0.77), 0.74 (95% CI = 0.60, 0.85), and 0.81 (95% CI = 0.72, 0.88). The pooled specificities for the IVC-CI, DIVCmax, DIVCmin, and carotid artery FTc were 0.82 (95% CI = 0.75, 0.87), 0.75 (95% CI = 0.66, 0.82), 0.76 (95% CI = 0.65, 0.84), and 0.87 (95% CI = 0.77, 0.93). The AUC for the IVC-CI, DIVCmax, DIVCmin, and carotid artery FTc were 0.84 (95% CI = 0.81, 0.87), 0.77 (95% CI = 0.73, 0.81), 0.82 (95% CI = 0.78, 0.85), and 0.91 (95% CI = 0.88, 0.93). Our study demonstrated that ultrasound indices are reliable predictors for PIH. The carotid artery FTc is probably the optimal ultrasound measurement for identifying patients who will develop PIH in our study.

## 1. Introduction

Postinduction hypotension (PIH) is very common in general anesthesia, with an incidence of 53% [1], and it is associated with many adverse outcomes [2,3,4]. An early study showed that the risk factors for PIH included elderly age, high scores of the American Society of Anesthesiologists (ASA) physical status, preexisting hypotension, use of propofol, and high fentanyl dose [5]. It has been confirmed in recent years that preoperative hypovolemia is closely associated with PIH [5,6]. The assessment and management of preoperative hypovolemia should be a key element for the prevention of PIH, but the accurate model to predict PIH is still unknown.

Many advanced monitoring methods are available to assess the fluid responsiveness and volume status, including pulse pressure variation (PPV), dynamic arterial elastance (Edyn), stroke volume variations (SVV), and measurements from ultrasound [7,8,9,10], but the former three are either invasive or limited under spontaneous breathing, and ultrasound is a safe, inexpensive, noninvasive, and real-time diagnostic technique with relatively low costs. Ultrasound was used before induction to evaluate the volume status and predict PIH in an increasing number of studies [9,10,11]. However, the results are conflicting, given the various monitored parameters from ultrasound in different studies [12,13,14].

Thus, a systematic review and meta-analysis was conducted in this study to evaluate the accuracy of the commonly used measurements from preoperative ultrasound to predict PIH in adult patients undergoing general anesthesia, and a meta-regression analysis was performed to test the accuracy of their availability.

## 2. Materials and Methods

### 2.1. Guidance for Conducting and Reporting

The methodology for conducting and reporting the systematic review followed the Preferred Reporting Items for Systematic Reviews and Meta-analyses of Diagnostic Test Accuracy Studies (PRISMA-DTA) guidelines [15]. This research was submitted to the International Prospective Register of Systematic Reviews (PROSPERO) on 2 January 2023, and study screening against eligibility criteria began on 9 January 2023. The registration number was CRD42023388622.

### 2.2. Eligibility Criteria

The inclusion criteria of the retrieved papers included those reporting adult patients (age > 18 years) undergoing general anesthesia and receiving either intubation or no intubation, observation trials, and randomized controlled trials, without language limitations. Case series, case reports, commentaries, letters, editorials, conference proceedings, abstracts, unpublished data, and studies not involving adult humans were excluded. The primary exposure was preoperative ultrasound measurement of the vasculature (including the inferior vena cava, subclavian vein, internal jugular vein, carotid artery, etc.) or cardiac chambers to assess the volume status.

### 2.3. Search Strategy

The PubMed, Cochrane Library, Embase, CNKI, and Web of Science databases were searched electronically from their inception to December 2023. The key terms were “postinduction”, “postintubation”, “induction”, “anesthesia induction”, “hypotension”, “low blood pressure”, and “ultrasound”, and various combinations of these terms were used. The search strategy is shown in Appendix A.

### 2.4. Study Selection and Data Extraction

Two researchers (LCY and AR) independently screened the titles and abstracts of all papers identified in the database search. Subsequently, they independently assessed the full text of the papers selected from the titles and abstracts screenings. The same investigators independently performed the data extraction. Any discrepancies during the selection process or data extraction were resolved by consensus or by the decision of a third independent researcher (LHL).

### 2.5. Assessment of Risk of Bias and Quality of the Evidence

Two trained investigators independently rated the quality of the selected studies. The quality assessment of diagnostic accuracy studies (QUADAS-2) tool was used to assess the risk of bias and applicability concerns in patient selection, index tests, reference standards, and flow and timing [16]. Each item was evaluated for a low, unclear, or high risk of bias [17].

### 2.6. Statistical Analysis

The statistical analyses were performed using metandi and midas in STATA (Stata Statistical Software 16), RevMan (version 5.3, Cochrane Collaboration, Oxford, UK), and Meta-disc. The bivariate model proposed by Reitsma et al.was used to assess the sensitivity and specificity of each index test for predicting PIH [18].

Only the index assessed in more than 5 studies was considered for quantitative summary receiver operating characteristic (SROC) analysis [19]. Meta-regression analysis was used to investigate the potential sources of heterogeneity in both sensitivity and specificity. Meta-Disc software was used to assess the threshold effect. The between-group (with or without PIH) difference was analyzed using the random-effect model and was expressed as mean ±SD. Continuous outcomes are presented using mean differences (MD). Heterogeneity was assessed using I2 value, and I2 > 50% was considered heterogeneity. Meta-regression was used to analyze potential sources of heterogeneity. Fagan plots were used to assess the clinical utility of the inferior vena cava collapsibility index (IVC-CI), Maximum inferior vena cava diameter (DIVCmax), Minimum inferior vena cava diameter (DIVCmin), and Carotid artery corrected flow time (FTc) for the diagnosis of PIH [20,21]. The possibility of publication bias was assessed by Deeks’ funnel plot [22]. A *p* value of <0.05 was considered statistical significance.

## 3. Results

### 3.1. Study Selection and Study Characteristics

**Figure 1:**
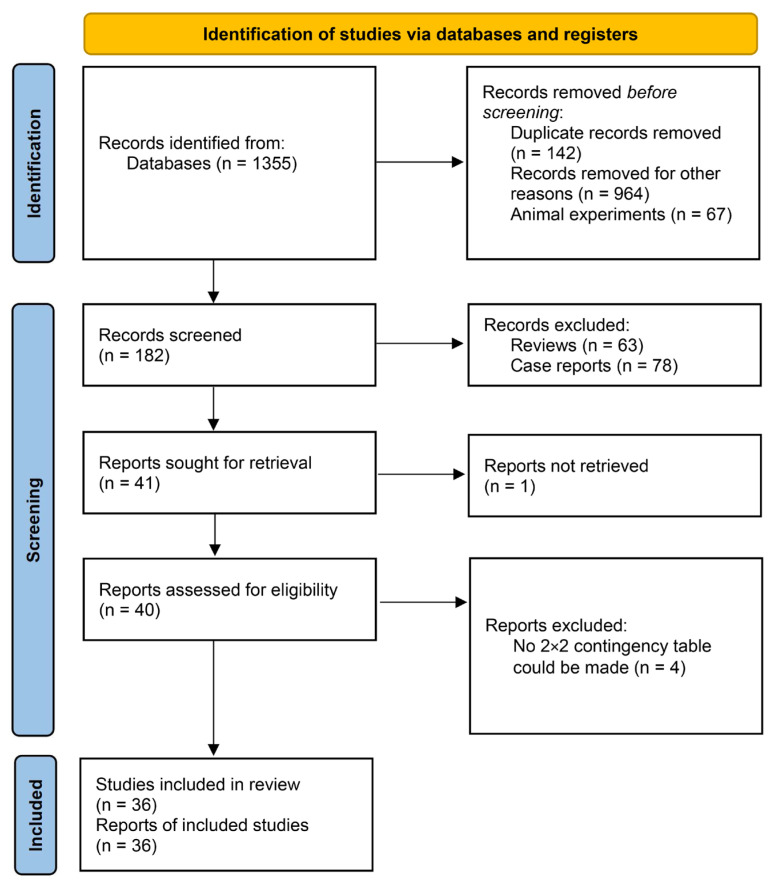
PRISMA flow diagram.

Our database search retrieved 1355 titles. After removing duplicates and other irrelevant studies, we screened the titles/abstracts of 41 records and assessed the full texts of 40 articles. Due to the available data lacking in four articles [23,24,25,26], thirty-six studies were ultimately included. The studies were published between 2016 and 2023. All studies included adult patients undergoing general anesthesia. The flow chart of the literature screening process is shown in Figure 1, and the study characteristics are shown in Table 1.

### 3.2. Inferior Vena Cava Collapsibility Index (IVC-CI)

**Figure 2:**
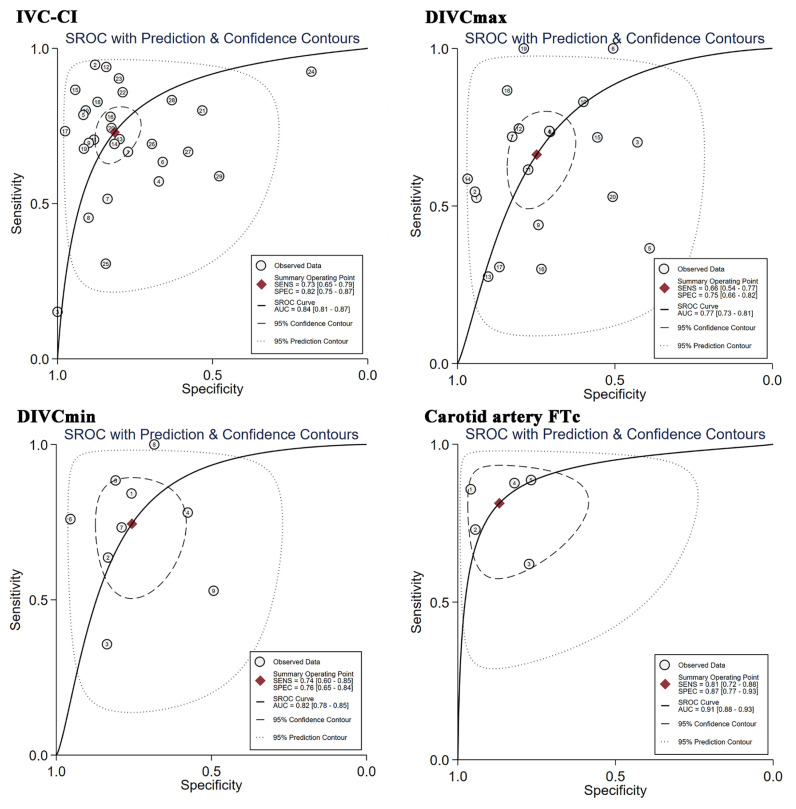
SROC curves.

The IVC-CI was reported in 27 studies [12,13,27,28,29,30,31,32,33,34,35,36,37,38,39,40,41,42,43,44,45,52,53,54,56,57,58] with 2467 patients. PIH was observed in 48.03% of the patients. The area under the receiver operating characteristic curve was 0.84 (95% CI = 0.81, 0.87) (Figure 2). The pooled sensitivity was 0.73 (95% CI = 0.65, 0.79), and the pooled specificity was 0.82 (95% CI = 0.75, 0.87) (Figure S1A). Substantial heterogeneity existed among the studies (*I*^2^ = 98%). The combined diagnostic odds ratio, positive likelihood ratio, and negative likelihood ratio were 12.00 (95% CI = 8.00, 19.00), 4.00 (95% CI = 3.00, 5.30), and 0.33 (95% CI = 0.26, 0.43), respectively (Table 2). Patients with PIH had higher IVC-CI values than those without PIH, with an MD of 10.47% (95% CI = 8.27, 12.67%, *p* < 0.001, *I*^2^ = 86%) (Figure S2A).

### 3.3. Maximum Inferior Vena Cava Diameter (DIVCmax)

DIVCmax was reported in 18 studies [12,13,27,28,29,31,33,34,37,38,39,40,42,43,44,45,57,58] that included 1654 patients. The area under the receiver operating characteristic curve was 0.77 (95% CI = 0.73, 0.81) (Figure 2). The pooled sensitivity was 0.66 (95% CI = 0.54, 0.77), and the pooled specificity was 0.75 (95% CI = 0.66, 0.82) (Figure S1B). Substantial heterogeneity exists among the studies (*I*^2^ = 99%). The combined diagnostic odds ratio, positive likelihood ratio, and negative likelihood ratio were 6.00 (95% CI = 3.00, 11.00), 2.70 (95% CI = 1.90, 3.70), and 0.45 (95% CI = 0.32, 0.63), respectively (Table 2). Patients with PIH had lower DIVCmax values than those without PIH, with an MD of −0.23 cm (95% CI = −0.30, −0.16 cm, *p* < 0.001, *I*^2^ = 86%) (Figure S2B).

### 3.4. Minimum Inferior Vena Cava Diameter (DIVCmin)

DIVCmin was reported in seven studies [34,37,42,44,45,57,58] that included 694 patients. The area under the receiver operating characteristic curve was 0.82 (95% CI = 0.78, 0.85) (*I*^2^ = 93%) (Figure 2). The pooled sensitivity was 0.74 (95% CI = 0.60, 0.85), and the pooled specificity was 0.76 (95% CI = 0.65, 0.84) (Figure S1C). The combined diagnostic odds ratio, positive likelihood ratio, and negative likelihood ratio were 9.00 (95% CI = 4.00, 21.00), 3.10 (95% CI = 2.00, 4.70), and 0.38 (95% CI = 0.25, 0.56), respectively (Table 2). Patients with PIH had lower DIVCmin values than those without PIH, with an MD of −0.28 cm (95% CI = −0.43, −0.12 cm, *p* = 0.001, *I*^2^ = 93%) (Figure S2C).

### 3.5. Carotid Artery Corrected Flow Time (FTc)

The carotid artery FTc was reported in five studies [14,47,50,55,59] that included 483 patients. The area under the receiver operating characteristic curve was 0.91 (95% CI = 0.88, 0.93) (*I*^2^ = 47%) (Figure 2). The pooled sensitivity was 0.81 (95% CI 0.72–0.88), and the pooled specificity was 0.87 (95% CI = 0.77, 0.93) (Figure S1D). The combined diagnostic odds ratio, positive likelihood ratio, and negative likelihood ratio were 29.00 (95% CI = 12.00, 67.00), 6.20 (95% CI = 3.40, 11.30), and 0.22 (95% CI = 0.14, 0.33), respectively (Table 2). Patients with PIH had lower carotid artery FTc values than those without PIH, with an MD of −31.52 ms (95% CI = −42.19, −20.86 ms, *p* = 0.001, *I*^2^ = 85%) (Figure S2D).

### 3.6. Fagan’s Nomograms

Fagan’s nomograms were generated to assess the clinical utility of the IVC-CI, DIVCmax, DIVCmin, and carotid artery FTc in diagnosing PIH at the population level (Figure S3A–D).Assuming a pre-test probability of 50%, Fagan’s nomogram showed that the post-test probability of PIH was 80%, 73%, 75%, and 86%, respectively, if the patients were diagnosed as positive from the IVC-CI, DIVCmax, DIVCmin, and carotid artery FTc. And the post-test probability of PIH was 25%, 31%, 25%, and 18%, respectively, if the patients were diagnosed as negative from the IVC-CI, DIVCmax, DIVCmin, and carotid artery FTc.

### 3.7. Other Ultrasound Measurements

SROC analysis was not performed for ultrasound measurements because there were fewer than five studies. The carotid artery respiratory variation of peak blood flow velocity was assessed in three studies [14,47,50]. IJV(internal jugular vein) was reported in four studies [36,37,49,51], but only two studies [37,49] measured IJV-area in Trendelenburg position, one study [37] measured IJV-area in supine position, one study [51] measured DIJV-CI in Trendelenburg position, one study [36] measured DIJV-CI in supine position, and one study [37] measured IJV change rate with posture (Trendelenburg and supine position). Four studies [27,45,46,52] recorded the SCV (subclavian vein). Only one study [30] measured the passive leg raising-induced changes in the velocity-time integral of the left ventricular outflow tract (DVTI-PLR). The carotid intima-media thickness was measured in one study [48]. The sensitivity and specificity of each of the above studies for the prediction of PIH are shown in Table 1.

### 3.8. Threshold Effect Analysis and Meta-Regression

The analysis using Meta-disc software revealed that the Spearman correlation coefficients between sensitivity and specificity of the IVC-CI, DIVCmax, DIVCmin, and carotid artery FTc were 0.016 (*p* = 0.935), 0.232 (*p* = 0.326), 0.233 (*p* = 0.546), and 0.300 (*p* = 0.624), respectively, which indicated that there were no threshold effects.

Figure S4A–D presented the results of the meta-regression for the sensitivity and specificity of the IVC-CI, DIVCmax, DIVCmin, and FTc. The meta-regression was used to analyze potential sources of heterogeneity, including the chosen cutoff, intubation or without intubation, type of opioid, use of etomidate, the median age > 60 years, and type of surgery (elective or emergency). The use of a cutoff higher than 50% and a median age higher than 60 years significantly reduced the sensitivity of the IVC-CI (*p* < 0.05). No tracheal intubation and no fentanyl significantly increased the sensitivity of DIVCmax (*p* < 0.05). The use of a cutoff higher than 340 ms and using etomidate for anesthesia induction reduced the sensitivity for carotid artery FTc (*p* < 0.001).

### 3.9. Publication Bias

Deeks’ funnel plots were used to evaluate publication bias in this meta-analysis. As shown in Figure S5A–D, the funnel plots showed symmetry, and the *p* values for the IVC-CI, DIVCmax, DIVCmin, and carotid artery FTc were 0.45, 0.81, 0.79, and 0.07, which indicated that there was no publication bias in this meta-analysis.

### 3.10. Risk of Bias and Quality of Evidence

The quality assessment of the studies is summarized in Figure S6. Almost all studies clearly stated that an ultrasound assessment was performed before anesthesia induction. The method of patient selection, whether consecutive or not, was not clearly reported in some studies [13,14,32,33,37,41,44,59]. There is also the presence of an unclear risk of bias in patient flow and timing because not all patients were analyzed due to poor ultrasound visualization, especially when examining the inferior vena cava.

## 4. Discussion

PIH can increase the risk of postoperative morbidity, including acute kidney injury and myocardial injury. Preoperative hypovolemia plays a key role in the development of PIH, and accurate assessment is critical for prevention. Our study showed that the AUC-SROCs of the IVC-CI, DIVCmax, DIVCmin, and carotid artery FTc from ultrasound were 0.84, 0.77, 0.82, and 0.91, respectively.

The area under the curve for carotid artery FTc was the largest among the four measurements, and their pooled sensitivity and specificity were 0.81 and 0.87, respectively, in our study. In previous studies [14,32,47,55,59], the sensitivity ranged from 0.61 to 0.89, and the specificity ranged from 0.77 to 0.94 (Table 1). The Fagan plot analysis [20,21] showed that when the pre-test probability was 50%, carotid artery FTc had an 86% probability of correctly detecting PIH following a positive measurement and lowering the probability of PIH to 18% when the measurement was negative. But the probability of a correct diagnosis rate did not exceed 80% for diagnosing PIH in the IVC-CI, DIVCmax, and DIVCmin. Compared with the IVC-CI, DIVCmax, and DIVCmin, the carotid artery FTc is probably more accurate for identifying PIH. The carotid artery is superficial, and little disturbed by spontaneous breathing [60]. The carotid artery FTc is decided by ventricular preload, cardiac contractility, and systemic vascular resistance (SVR) [61]. One earlier meta-analysis [10] has shown that the carotid artery FTc has a high diagnostic accuracy for the prediction of PIH and fluid responsiveness. In our study, the result was similar, and the emergency surgery did not affect the accuracy of carotid artery FTc. Some of the included articles found [37,38,40,41] that the carotid artery FTc was reliable in predicting PIH, except one [14], which presented opposite findings. Two studies set cutoff values > 340 ms, which affected the sensitivity from meta-regression [14,47]. One included elderly patients without hypertension; the other included patients with peritonitis. The longer duration of carotid artery FTc in the former may be due to the slower heart rate in elderly individuals, resulting in a relatively prolonged duration of left ventricular contraction [62]. The latter may be due to the effect of generalized systemic inflammation on ventricular preload, cardiac contractility, and systemic vascular resistance.

The IVC is determined by right atrial pressure, intra-abdominal pressure, and intravascular volume [63]. The IVC varies during the respiratory cycle, with a minimum end-inspiratory diameter and a maximum end-expiratory diameter occurring during spontaneous breathing [64]. A previous review [9] reported that preoperative measurement of the IVC-CI using ultrasound can predict PIH. In our study, we focused on the ability of the IVC to predict PIH and used meta-Disc software to assess the threshold effects of the IVC-CI, DIVCmax, and DIVCmin, which showed no threshold effects for them. By pooling data, we found that the IVC-CI was more accurate than DIVCmax and DIVCmin. However, it is obvious that IVC has a high rate of measurement failure because of obesity, tissue edema, gastrointestinal gas accumulation, or abdominal trauma, whereas the carotid artery FTc is accessible in all patients.

The results of our study showed that the cutoff and age > 60 years were associated with the accuracy of the IVC-CI, and meta-regression analysis revealed a significant decrease in sensitivity for the prediction of PIH when the cutoff of the IVC-CI exceeded 50%, which was consistent with the results from previous studies [13,44]. Several studies [65,66,67] have shown that the baseline inferior vena cava (IVC) diameter varies with age, gender, weight, body mass index (BMI), and body surface area. In the future, the cutoff values could be personalized based on patient characteristics. Meta-regression showed that no tracheal intubation or no fentanyl for anesthesia induction affected the sensitivity of the DIVCmax. Two articles [42,57] of patients were included in our study who underwent colonoscopy without tracheal intubation. Without liquid infusion during the examination and the use of propofol induction in all patients, the sensitivity of DIVCmax for the prediction of PIH may improve. Three studies [29,42,57] did not use fentanyl for the induction of anesthesia, and all patients underwent bowel preparation, which differs from other studies. This difference may have affected the accuracy of DIVCmax.

This study has a few limitations. Some of the studies had relatively small sample sizes, which could reduce the statistical power and the ability to detect significant associations. Second, the definitions of PIH were not consistent among the included studies, which might make it challenging to analyze findings across studies. And the differences in cutoff values for each parameter in the included studies were inconsistent. Third, our study focused solely on the use of ultrasound to predict PIH and did not analyze the use of other modalities. Fourth, The type of hypnotic used and the speed of injection play a role in predicting postinduction hypotension. Fifth, The effect of operator experience on the reliability of ultrasound interpretation may influence the results. Sixth, this result of carotid artery FTc does not apply to patients with peripheral arterial diseases and atherosclerosis because the Doppler signal may be altered. Finally, not all patients were analyzed due to poor ultrasound visualization when examining IVC, which led to an unclear risk of bias in patient flow and timing, and these issues might affect the accuracy of the results.

## 5. Conclusions

In conclusion, preoperative ultrasound measurements of the inferior vena cava and carotid artery FTc can predict PIH, and the carotid artery FTc is probably more accurate for identifying patients who will develop PIH. Age > 60 years significantly affects the accuracy of the IVC-CI, and the cutoff might affect the accuracy of the IVC-CI and carotid artery FTc. Moreover, no tracheal intubation or fentanyl for anesthesia induction would affect the accuracy of the DIVCmax.

## Figures and Tables

**Table 1:** Study characteristics.

Author	Year	Age (Years)	Definition of PIH	Drugs	US Index	Cutoff	TP	FP	TN	FN	SE (95% CI)	SP (95% CI)
Rose N et al. [27]	2022	≥18	MAP < 60 mmHg Or > 30% decrease	Fentanyl Propofol	IVC-CI	37%	47	11	59	3	0.94 (0.83–0.99)	0.84 (0.74–0.92)
					DIVCmax	1.97 cm	22	18	52	28	0.44 (0.30–0.59)	0.74 (0.62–0.84)
					DSCVmax ^1^	0.69 cm	44	35	35	6	0.88 (0.76–0.95)	0.50 (0.38–0.62)
					DSCVmax ^2^	0.7 cm	44	39	31	6	0.88 (0.76–0.95)	0.44 (0.32–0.57)
					DSCV-CI ^1^	23.4%	32	16	54	18	0.64 (0.49–0.77)	0.77 (0.66–0.86)
					DSCV-CI ^2^	36%	45	9	61	5	0.90 (0.78–0.97)	0.87 (0.77–0.94)
Amin SR et al. [28]	2022	>60	MAP < 60 mmHg or >30% decrease	Fentanyl Propofol	IVC-CI	33.6%	27	9	40	12	0.69 (0.52–0.83)	0.82 (0.68–0.91)
					DIVCmax	1.63 cm	24	11	38	15	0.62 (0.45–0.77)	0.78 (0.63–0.88)
Sari S et al. [29]	2019	≥18	MAP < 60 mmHg or >30% decrease	Propofol Remifentanil	IVC-CI	32.8%	29	7	38	10	0.74 (0.58–0.87)	0.84 (0.71–0.94)
					DIVCmax	1.58 cm	28	20	25	11	0.72 (0.55–0.85)	0.56 (0.40–0.70)
Turoni L et al. [12]	2022	66 ± 9	MAP < 60 mmHg	Fentanyl Etomidate	IVC-CI	49.5%	8	21	24	2	0.80 (0.44–0.97)	0.53 (0.38–0.68)
					DIVCmax	1.54 cm	3	12	33	7	0.30 (0.07–0.65)	0.73 (0.58–0.85)
Aissaoui Y et al. [30]	2022	>50	MAP < 65 mmHg or >30% decrease SBP < 90 mmHg or >30% decrease	Fentanyl Propofol	IVC-CI	42%	17	5	26	16	0.52 (0.34–0.69)	0.84 (0.66–0.95)
					ΔVTI-PLR	18%	29	5	26	4	0.88 (0.72–0.97)	0.84 (0.66–0.95)
Qiu XS et al. [31]	2020	≥18	MAP < 65 mmHg or >20% decrease	Fentanyl Propofol	IVC-CI	42.1%	24	6	45	10	0.71 (0.53–0.85)	0.88 (0.76–0.96)
					DIVCmax	1.66 cm	25	15	36	9	0.74 (0.56–0.87)	0.71 (0.56–0.83)
Szabo M et al. [32]	2019	≥18	SBP < 90 mmHg or >30% decrease	Fentanyl Propofol	IVC-CI	50%	15	5	45	18	0.45 (0.28–0.64)	0.90 (0.78–0.97)
Bhimsaria SK et al. [33]	2022	≥18	MAP > 20% decrease	Fentanyl Propofol	IVC-CI	50%	46	7	28	19	0.71 (0.58–0.81)	0.80 (0.63–0.92)
					DIVCmax	1.3 cm	54	14	21	11	0.83 (0.72–0.91)	0.60 (0.42–0.76)
Goyal A et al. [34]	2022	≥18	MAP < 60 mmHg or >30% decrease	Fentanyl	IVC-CI	37.5%	26	20	39	15	0.63 (0.47–0.78)	0.66 (0.53–0.78)
					DIVCmax	1.38 cm	15	36	23	26	0.37 (0.22–0.53)	0.39 (0.27–0.53)
					DIVCmin	0.94 cm	32	25	34	9	0.78 (0.62–0.89)	0.58 (0.44–0.70)
Au AK et al. [35]	2016	≥18	SBP < 90 mmHg	Propofol	IVC-CI	50%	12	5	17	6	0.67 (0.41–0.87)	0.77 (0.55–0.92)
Cao Y et al. [36]	2021	45–60	MAP < 60 mmHg or >20% decrease	Fentanyl Etomidate	IVC-CI	39.3%	44	4	43	21	0.68 (0.55–0.79)	0.91 (0.80–0.98)
					IJV-CI (supine)	40.04%	46	5	42	19	0.71 (0.58–0.81)	0.89 (0.77–0.96)
Khaled D et al. [37]	2023	≥18	MAP > 20% decrease	Fentanyl Propofol	IVC-CI	36.3%	48	16	33	36	0.57 (0.46–0.68)	0.67 (0.52–0.80)
					DIVCmax	1.73 cm	59	28	21	25	0.70 (0.59–0.80)	0.43 (0.29–0.58)
					DIVCmin	0.84 cm	30	8	41	54	0.36 (0.26–0.47)	0.84 (0.70–0.93)
					IJV-A(supine)	14.4 mm^2^	69	29	20	15	0.82 (0.72–0.90)	0.41 (0.27–0.56)
					IJV-A (Trendelenburg)	17.4 mm^2^	59	30	19	25	0.70 (0.59–0.80)	0.39 (0.25–0.54)
					IJV change rate	28%	46	12	37	38	0.55 (0.44–0.66)	0.76 (0.61–0.87)
He FJ et al. [38]	2022	≥18	MAP < 60 mmHg or >20% decrease	Fentanyl Etomidate	IVC-CI	42.5%	22	1	40	8	0.73 (0.54–0.88)	0.98 (0.87–1.00)
					DIVCmax	1.85 cm	8	4	37	22	0.27 (0.12–0.46)	0.90 (0.77–0.97)
Li GF et al. [39]	2020	≥18	MAP < 60 mmHg or >25% decrease	Fentanyl Propofol	IVC-CI	34%	46	7	34	13	0.78 (0.65–0.88)	0.83 (0.68–0.93)
					DIVCmax	1.62 cm	44	8	33	15	0.75 (0.62–0.85)	0.80 (0.65–0.91)
Cheng SS et al. [40]	2020	50–80	MAP < 60 mmHg or >20% decrease	Fentanyl Propofol	IVC-CI	42%	24	4	27	5	0.83 (0.64–0.94)	0.87 (0.70–0.96)
					DIVCmax	1.7 cm	17	1	30	12	0.59 (0.39–0.76)	0.97 (0.83–1.00)
Purshothaman SS et al. [41]	2020	≥18	MAP < 60 mmHg	Fentanyl Propofol	IVC-CI	43%	13	2	33	2	0.87 (0.60–0.98)	0.94 (0.81–0.99)
Duan FY et al. [42]	2021	≥18	MAP < 60 mmHg or >20% decrease	Propofol	IVC-CI	31.9%	20	2	20	5	0.80 (0.59–0.93)	0.91 (0.71–0.99)
					DIVCmax	1.65 cm	18	3	19	7	0.72 (0.51–0.88)	0.86 (0.65–0.97)
					DIVCmin	1.15 cm	19	1	21	6	0.76 (0.55–0.91)	0.95 (0.77–1.00)
Zhang J et al. [43]	2016	≥18	MAP < 60 mmHg or >30% decrease	Fentanyl Etomidate	IVC-CI	43%	33	4	44	9	0.79 (0.63–0.90)	0.92 (0.80–0.98)
					DIVCmax	1.8 cm	31	14	34	11	0.74 (0.58–0.86)	0.71 (0.56–0.83)
Zhang HY et al. (a) [44]	2022	≥18	MAP < 60 mmHg or >30% decrease	Fentanyl Etomidate	IVC-CI	43%	18	4	29	1	0.95 (0.74–1.00)	0.88 (0.72–0.97)
					DIVCmax	1.29 cm	10	2	31	9	0.53 (0.29–0.76)	0.94 (0.80–0.99)
					DIVCmin	0.88 cm	16	8	25	3	0.84 (0.60–0.97)	0.76 (0.58–0.89)
Zhang HY et al. (b) [44]	2022	≥18	MAP < 60 mmHg or >30% decrease	Fentanyl Etomidate	IVC-CI	50%	5	0	18	28	0.15 (0.05–0.32)	1.00 (0.81–1.00)
					DIVCmax	1.24 cm	18	1	17	15	0.55 (0.36–0.72)	0.94 (0.73–1.00)
					DIVCmin	0.88 cm	21	3	15	12	0.64 (0.45–0.80)	0.83 (0.59–0.96)
Zheng DQ et al. [45]	2023	42–73	MAP < 60 mmHg or >20% decrease	Fentanyl Propofol	IVC-CI	40.9%	48	9	80	21	0.70 (0.57–0.80)	0.90 (0.82–0.95)
					DIVCmax	2 cm	69	44	45	0	1.00 (0.95–1.00)	0.51 (0.40–0.61)
					DIVCmin	1.16 cm	61	17	72	8	0.88 (0.78–0.95)	0.81 (0.71–0.88)
					DSCVmax ^1^	0.86 cm	61	30	59	8	0.88 (0.78–0.95)	0.66 (0.55–0.76)
					DSCVmin ^1^	0.57 cm	48	4	85	21	0.70 (0.57–0.80)	0.96 (0.89–0.99)
					DSCV-CI ^1^	33%	59	54	35	10	0.86 (0.75–0.93)	0.39 (0.29–0.50)
Yang LJ et al. [46]	2023	≥18	MAP < 60 mmHg or >30% decrease	Sufentanil Propofol	ΔDSC	15.86%	15	7	73	12	0.56 (0.35–0.75)	0.91 (0.83–0.96)
Wang J et al. [47]	2022	65–75	MAP < 65 mmHg or >20% decrease	Sufentanil Etomidate	Carotid artery FTc	379.1 ms	46	2	34	18	0.72 (0.59–0.82)	0.94 (0.81–0.99)
					Carotid artery ΔVpeak	7.5%	35	9	27	28	0.56 (0.42–0.68)	0.75 (0.58–0.88)
Kaydu A et al. [48]	2019	≥18	MAP > 20% decrease	Fentanyl propofol	CIMT	0.65 mm	31	10	29	10	0.76 (0.60–0.88)	0.74 (0.58–0.87)
Okamura K et al. [49]	2019	≥18	MAP < 60 mmHg or >30% decrease	Fentanyl Propofol	IJV-A (Trendelenburg)	1.48 cm^2^	28	25	20	9	0.76 (0.59–0.88)	0.44 (0.30–0.66)
Maitra S et al. [50]	2020	≥18	MAP < 65 mmHg or >20% decrease SBP < 90 mmHg or >30% decrease	Fentanyl Propofol	Carotid artery FTc	330.2 ms	54	2	47	9	0.86 (0.75–0.93)	0.96 (0.86–1.00)
					Carotid artery ΔVpeak	18.8%	39	16	33	24	0.62 (0.49–0.74)	0.67 (0.52–0.80)
Kilic Y et al. [51]	2020	≥18	MAP < 65 mmHg or >20% decrease	Remifentanil	IJV-CI (Trendelenburg)	19.9%	15	6	10	9	0.63 (0.41–0.81)	0.63 (0.35–0.85)
Chowdhury AR et al. [14]	2023	≥18	MAP < 65 mmHg or >20% decrease SBP < 90 mmHg or >30% decrease	Fentanyl Etomidate	Carotid artery FTc	344.8 ms	18	7	24	11	0.62 (0.42–0.79)	0.77 (0.59–0.90)
					Carotid artery ΔVpeak	7.9%	18	14	17	11	0.62 (0.42–0.79)	0.55 (0.36–0.73)
Chen HJ et al. [52]	2023	65–95	MAP < 60 mmHg or >20% decrease	Etomidate Sufentanil	IVC-CI	36.6%	79	17	64	13	0.86 (0.77–0.92)	0.79 (0.69–0.87)
					DSCV-CI ^1^	31.25%	66	8	73	26	0.72 (0.61–0.81)	0.90 (0.81–0.96)
FathyMM et al. [53]	2023	21–70	MAP < 65 mmHg SBP > 30%decrease	Fentanyl Propofol	IVC-CI	39%	55	19	72	7	0.89 (0.78–0.95)	0.79 (0.69–0.87)
Omar H et al. [54]	2023	≥18	MAP < 60 mmHg or >20% decrease	Fentanyl Propofol	IVC-CI	28.3%	74	18	4	6	0.93 (0.84–0.97)	0.18 (0.05–0.40)
					DIVCmax/(Ao ratio index)	0.852	62	8	14	18	0.78 (0.67–0.86)	0.64 (0.41–0.83)
Huang SS et al. [55]	2023	65–85	MAP < 60 mmHg or >20% decrease SBP < 90 mmHg or >30% decrease	Sufentanil Propofol	Carotid artery FTc	334.95 ms	64	7	32	9	0.88 (0.78–0.94)	0.82 (0.66–0.92)
Agarwal J et al. [13]	2022	≥18	MAP < 65 mmHg SBP < 90 mmHg or >25% decrease	Fentanyl Propofol	IVC-CI	63.35%	19	7	38	43	0.31 (0.20–0.44)	0.84 (0.71–0.94)
					DIVCmax	1.14 cm	19	6	39	43	0.31 (0.20–0.44)	0.87 (0.73–0.95)
Jaya W et al. [56]	2021	15–64	MAP > 30% decrease	Fentanyl propofol	IVC-CI	62.7%	9	7	16	4	0.69 (0.39–0.91)	0.70 (0.47–0.87)
					CAo-I	85.55%	6	12	11	7	0.46 (0.19–0.75)	0.48 (0.27–0.69)
Xu QQ et al. (a) [57]	2021	60–80	SBP > 30% decrease	Sufentanil Propofol								
					DIVCmax	1.25 cm	13	3	16	2	0.87 (0.60–0.98)	0.84 (0.60–0.97)
					DIVCmin	0.78 cm	11	4	15	4	0.73 (0.45–0.92)	0.79 (0.54–0.94)
Xu QQ et al. (b) [57]	2021	60–80	SBP > 30% decrease	Sufentanil Propofol	IVC-CI	37%	10	7	12	2	0.83 (0.52–0.98)	0.63 (0.38–0.84)
					DIVCmax	1.23 cm	12	4	15	0	1.00 (0.74–1.00)	0.79 (0.54–0.94)
					DIVCmin	0.82 cm	12	6	13	0	1.00 (0.74–1.00)	0.68 (0.43–0.87)
Mohammed S et al. [58]	2021	≥18	MAP < 65 mmHg or >30% decrease	Fentanyl Propofol	IVC-CI	46%	10	37	34	7	0.59 (0.33–0.82)	0.48 (0.36–0.60)
					DIVCmax	1.42 cm	9	35	36	8	0.53 (0.28–0.77)	0.51 (0.39–0.63)
					DIVCmin	0.73 cm	9	36	35	8	0.53 (0.28–0.77)	0.49 (0.37–0.61)
Yang Y et al. [59]	2023	≥18	MAP < 60 mmHg or >30% decrease	Sufentanil Propofol	Carotid artery FTc	335.83 ms	39	13	43	5	0.89 (0.75–0.96)	0.77 (0.64–0.87)
					IVC-CI	39%	10	8	11	5	0.67 (0.38–0.88)	0.58 (0.33–0.80)

Notes: ^1^: spontaneous inspiration breathing; ^2^: deep inspiration breathing; ASA: American Society of Anesthesiologists; PIH: post-induction hypotension; US: ultrasound; TP: true positive; FP: false positive; TN: true negative; FN: false negative; SE: sensitivity; SP: specificity; IVC: Inferior vena cava; CI: collapsibility index; DIVCmax: The maximum diameters of inferior vena cava; SCV: subclavian vein; DSCVmax: The maximum diameters of SCV; DSCV-CI: The collapsibility index of SCV; ΔVTI-PLR: velocity-time integral of the left ventricular outflow tract; DIVCmin: The minimum diameters of inferior vena cava; IJV: internal jugular vein; IJV-A: The area of internal jugular vein; FTc carotid artery corrected flow time; Carotid artery ΔVpeak: respiratory variation of peak blood flow velocity in the common carotid artery; CIMT: carotid intima–media thickness; Ao ratio index: caval aorta index; CAo-I: caval aortic index.

**Table 2:** Diagnostic Test Accuracy Results.

Index	Test	N	Positive Likelihood Ratio (95% CI)	Negative Likelihood Ratio (95% CI)	Diagnostic Odds Ratio (95% CI)
IVC-CI	29	2467	4.0 (3.0, 5.3)	0.33 (0.26, 0.43)	12 (8, 19)
DIVCmax	20	1654	2.7 (1.9, 3.7)	0.45 (0.32, 0.63)	6 (3, 11)
DIVCmin	9	694	3.1 (2.0, 4.7)	0.34 (0.20, 0.56)	9 (4, 21)
Carotid artery FTc	5	483	6.2 (3.4, 11.3)	0.22 (0.14, 0.33)	29 (12, 67)

Data reported as estimate value (95% CI). Abbreviations: IVC-CI: collapsibility index of inferior vena cava; DIVCmax: maximum diameters of inferior vena cava; DIVCmin: minimum diameters of inferior vena cava; Carotid artery FTc: carotid artery corrected flow time.

## Data Availability

The data presented in this study are available on request from the corresponding author.

## Supplementary Materials

The following supporting information can be downloaded at: https://www.mdpi.com/article/10.3390/jpm14050452/s1, Figure S1A: Forest plot for sensitivity and specificity of IVC-CI for diagnosis of PIH; Figure S1B: Forest plot for sensitivity and specificity of DIVCmax for diagnosis of PIH; Figure S1C: Forest plot for sensitivity and specificity of DIVCmin for diagnosis of PIH; Figure S1D: Forest plot for sensitivity and specificity of carotid artery FTc for diagnosis of PIH; Figure S2A: Forest plot for the mean difference of IVC-CI between patients with PIH and without PIH; Figure S2B: Forest plot for the mean difference of DIVCmax between patients with PIH and without PIH; Figure S2C: Forest plot for the mean difference of DIVCmin between patients with PIH and without PIH; Figure S2D: Forest plot for the mean difference of carotid artery FTc between patients with PIH and without PIH; Figure S3A: Fagan’s nomogram for IVC-CI; Figure S3B: Fagan’s nomogram for DIVCmax; Figure S3C: Fagan’s nomogram for DIVCmin; Figure S3D: Fagan’s nomogram for carotid artery FTc; Figure S4A: Meta-regression for IVC-CI; Figure S4B: Meta-regression for DIVCmax; Figure S4C: Meta-regression for DIVCmin; Figure S4D: Meta-regression for carotid artery FTc; Figure S5A: Deeks’ funnel plot asymmetry test for publication bias of IVC-CI; Figure S5B: Deeks’ funnel plot asymmetry test for publication bias of DIVCmax; Figure S5C: Deeks’ funnel plot asymmetry test for publication bias of DIVCmin; Figure S5D: Deeks’ funnel plot asymmetry test for publication bias of carotid artery FTc; Figure S6: Assessment of Risk of Bias According to QUADAS-2.

## Appendix A. Search Strategies

#1. IVC
#2. “(vena cava, inferior)”[MeSH Terms] OR (Inferior vena cava) OR (Inferior Vena Cavas) OR (Vena Cavas, Inferior)
#3. IVC collapsibility index
#4. IVCCI
#5. Inferior vena cava diameter
#6. IVC variability
#7. IVC distensibility
#8. IVC collapsibility
#9. IVC spontaneous breathing
#10. IVCD
#11. #1 OR #2 OR #3 OR #4 OR #5 OR #6 OR #7 OR #8 OR #9 OR #10
#12. “(carotid arteries)”[MeSH Terms] OR (carotid artery) OR (Arteries, Carotid) OR (Artery, Carotid)
#13. internal jugular vein
#14. IJV
#15. IJV-area
#16. internal jugular vein area
#17. IJV collapsibility index
#18. IJVCI
#19. internal jugular vein diameter
#20. IJV variability
#21. IJV distensibility
#22. IJV collapsibility
#23. IJVD
#24. #13 OR #14 OR #15 OR #16 OR #17 OR #18 OR #19 OR #20 OR #21 OR #22 OR #23
#25. “(subclavian vein)”[MeSH Terms] OR (subclavian vein) OR (Subclavian Veins) OR (Vein, Subclavian) OR (Veins, Subclavian)
#26. SCV
#27. SCV collapsibility index
#28. SCVCI
#29. subclavian vein diameter
#30. SCV variability
#31. SCV distensibility
#32. SCV collapsibility
#33. SCVD
#34. #25 OR #26 OR #27 OR #28 OR #29 OR #30 OR #31 OR #32 OR #33
#35. “echocardiography”[MeSH Terms] OR Echocardiography OR (cardiac ultrasound)
#36. “(blood vessels)”[MeSH Terms] OR (Blood Vessel) OR (Vessel, Blood) OR (Vessels, Blood)
#37. “arteries”[MeSH Terms] OR Artery
#38. “veins”[MeSH Terms] OR Vein
#39. #35 OR #36 OR #37 OR #38
#40. #11 OR #24 OR #34 OR #39
#41. “(diagnostic imaging)”[ MeSH Terms] OR “ultrasonography”[MeSH Terms] OR “ultrasonic”[MeSH Terms] OR Ultrasound OR Ultrasound-guided OR Sonography OR Echography OR Echotomography OR Ultrasonic
#42. “hypotension”[MeSH Terms] OR hypotension OR (Vascular Hypotension) OR (Low Blood Pressure) OR (Blood Pressure, Low) OR (Hypotension, Vascular)
#43. “(anesthesia, general)”[MeSH Terms] OR general anesthesia
#44. Postinduction
#45. Post-induction
#46. Postintubation
#47. Post-intubation
#48. propofol induction
#49. anesthesia induction
#50. #43 OR #44 OR #45 OR #46 OR #47 OR #48 OR #49
#51. #40 AND #41 AND #42 AND #50
