# Supplementary material for: Preoperative Ultrasound for the Prediction of Postinduction Hypotension: A Systematic Review and Meta-Analysis
Source: J Pers Med. 2024 Apr 25;14(5):452. doi: 10.3390/jpm14050452 (PMC11122148; doi:10.3390/jpm14050452)
Supplement: Supplementary file 1 [file jpm-14-00452-s001.zip › Appendix A.pdf]

Appendix A: Search strategies

#1. IVC

#2. "(vena cava, inferior)"[MeSH Terms] OR (Inferior vena cava) OR (Inferior Vena Cavas) OR (Vena Cavas, Inferior)

#3. IVC collapsibility index

#4. IVCCI

#5. Inferior vena cava diameter

#6. IVC variability

#7. IVC distensibility

#8. IVC collapsibility

#9. IVC spontaneous breathing

#10. IVCD

#11. #1 OR #2 OR #3 OR #4 OR #5 OR #6 OR #7 OR #8 OR #9 OR #10

#12. "(carotid arteries)"[MeSH Terms] OR (carotid artery) OR (Arteries, Carotid) OR (Artery, Carotid)

#13. internal jugular vein

#14. IJV

#15. IJV-area

#16. internal jugular vein area

#17. IJV collapsibility index

#18. IJVCI

#19. internal jugular vein diameter

#20. IJV variability

#21. IJV distensibility

#22. IJV collapsibility

#23. IJVD

#24. #13 OR #14 OR #15 OR #16 OR #17 OR #18 OR #19 OR #20 OR #21 OR #22 OR #23

#25. "(subclavian vein)"[MeSH Terms] OR (subclavian vein) OR (Subclavian Veins) OR (Vein, Subclavian) OR (Veins, Subclavian)

#26. SCV

#27. SCV collapsibility index

#28. SCVCI

#29. subclavian vein diameter

#30. SCV variability

#31. SCV distensibility

#32. SCV collapsibility

#33. SCVD

#34. #25 OR #26 OR #27 OR #28 OR #29 OR #30 OR #31 OR #32 OR #33

#35. "echocardiography"[MeSH Terms] OR Echocardiography OR (cardiac ultrasound)

#36. "(blood vessels)"[MeSH Terms] OR (Blood Vessel) OR (Vessel, Blood) OR (Vessels, Blood)

#37. "arteries"[MeSH Terms] OR Artery

#38. "veins"[MeSH Terms] OR Vein

#39. #35 OR #36 OR #37 OR #38

#40. #11 OR #24 OR #34 OR #39

#41. "(diagnostic imaging)"[MeSH Terms] OR "ultrasonography"[MeSH Terms] OR "ultrasonic"[MeSH Terms] OR Ultrasound OR Ultrasound-guided OR Sonography OR Echography OR Echotomography OR Ultrasonic

#42. "hypotension"[MeSH Terms] OR hypotension OR (Vascular Hypotension) OR (Low Blood Pressure) OR (Blood Pressure, Low) OR (Hypotension, Vascular)

#43. "(anesthesia, general)"[MeSH Terms] OR general anesthesia

#44. Postinduction

#45. Post-induction

#46. Postintubation

#47. Post-intubation

#48. propofol induction

#49. anesthesia induction

#50. #43 OR #44 OR #45 OR #46 OR #47 OR #48 OR #49

#51. #40 AND #41 AND #42 AND #50
