# Supplementary material for: Preoperative Ultrasound for the Prediction of Postinduction Hypotension: A Systematic Review and Meta-Analysis
Source: J Pers Med. 2024 Apr 25;14(5):452. doi: 10.3390/jpm14050452 (PMC11122148; doi:10.3390/jpm14050452)
Supplement: Supplementary file 1 [file jpm-14-00452-s001.zip › Supplementary Materials.pdf]

Supplementary Materials

Figure S1A: Forest plot for sensitivity and specificity of IVC-CI for diagnosis of PIH.

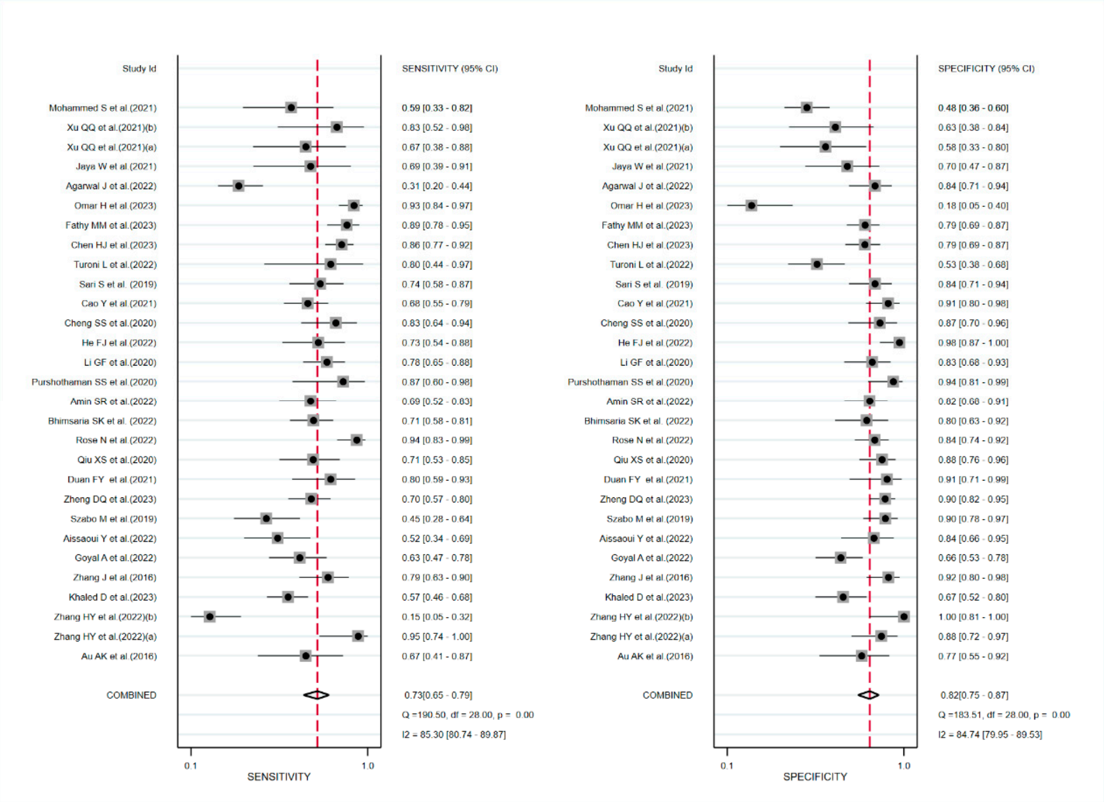

Figure S1B: Forest plot for sensitivity and specificity of DIVCmax for diagnosis of PIH.

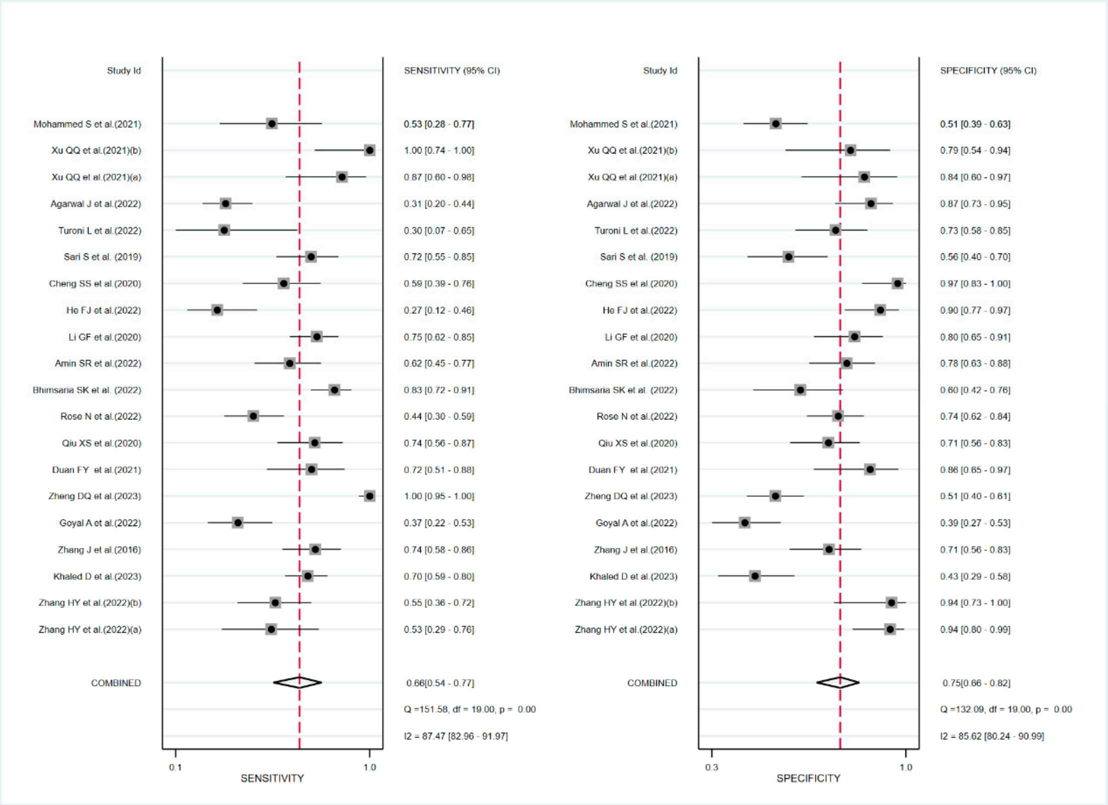

Figure S1C: Forest plot for sensitivity and specificity of DIVCmin for diagnosis of PIH.

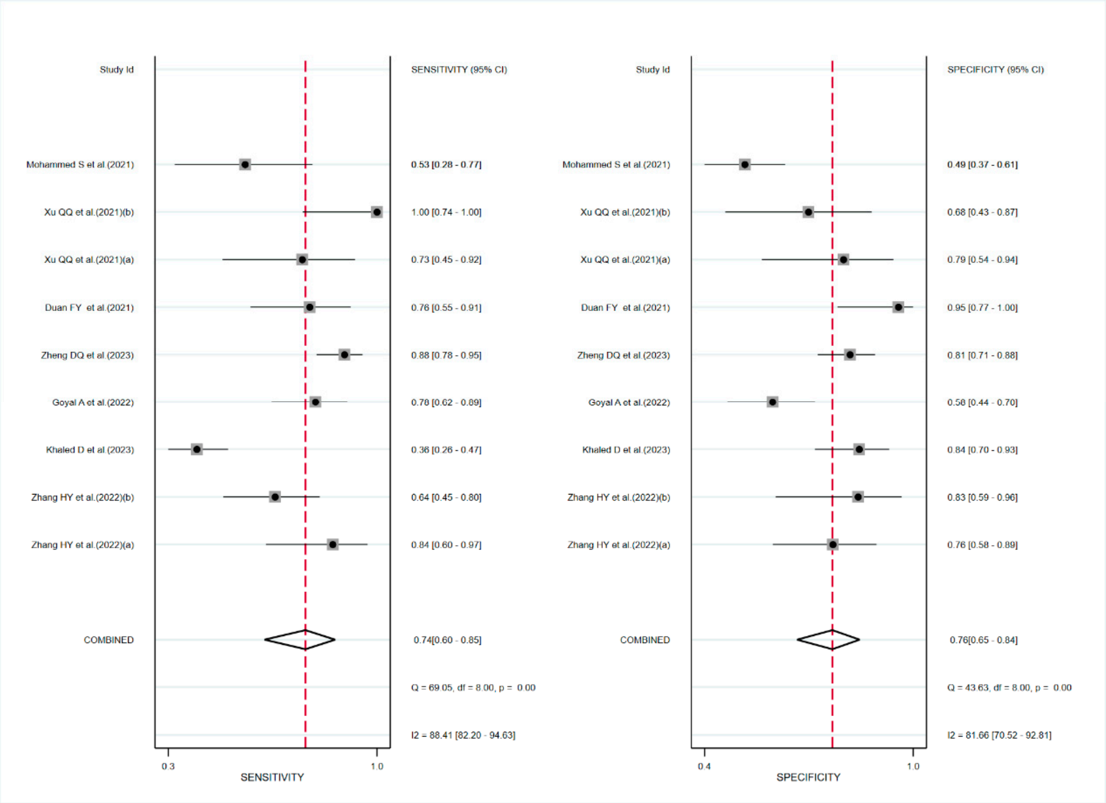

Figure S1D: Forest plot for sensitivity and specificity of carotid artery FTc for diagnosis of PIH.

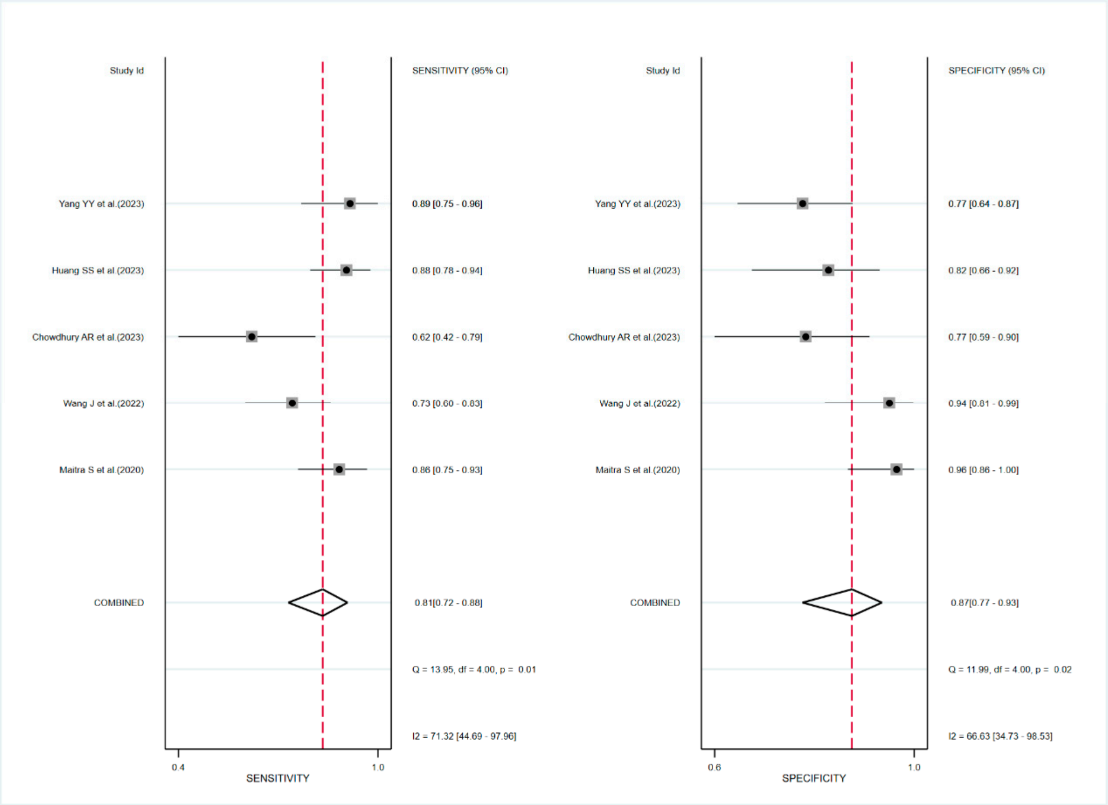

Figure S2A: Forest plot for the mean difference of IVC-CI between patients with PIH and without PIH.

Study or Subgroup	PIH Mean	PIH SD	PIH Total	NO PIH Mean	NO PIH SD	NO PIH Total	Weight	Mean Difference IV, Random, 95% CI
Aissaoui Y et al. 2022	39.8	19.37	33	30.43	17.1	31	2.9%	9.37 [0.43, 18.31]
Au AK et al. 2016	44	19.1	18	38	16.92	22	2.3%	6.00 [-5.31, 17.31]
Bhimsaria SK et al. 2022	55	5.58	65	46.93	5.4	35	5.2%	8.07 [5.82, 10.32]
Cao Y et al. 2021	42	10	47	26	11	65	4.7%	16.00 [12.09, 19.91]
Chen HJ et al. 2023	43.22	8.54	92	29.69	8.2	81	5.2%	13.53 [11.03, 16.03]
Cheng et al. 2020	46.6	4.6	29	38.2	3.8	31	5.3%	8.40 [6.26, 10.54]
Duan FY et al. 2021	39.05	8.56	25	26.48	4.08	22	4.8%	12.57 [8.81, 16.33]
Fathy MM et al. 2023	49	8	62	33	8	91	5.1%	16.00 [13.42, 18.58]
Goyal A et al. 2022	43.6	18.6	41	33.1	13.6	59	3.7%	10.50 [3.83, 17.17]
He FJ et al. 2022	45.1	8.1	30	32	6.7	41	4.8%	13.10 [9.55, 16.65]
Jaya W et al. 2021	61.27	16.99	13	48.31	20.74	23	2.0%	12.96 [0.42, 25.50]
Khaled D et al. 2023	40	15.28	84	30	14.81	49	4.2%	10.00 [4.72, 15.28]
Li GF et al. 2020	45	14	59	28	12	41	4.3%	17.00 [11.88, 22.12]
Mohammed S et al. 2021	47	15	17	47	14	71	3.3%	0.00 [-7.84, 7.84]
Omar H et al. 2023	44.5	9.8	80	43.8	11.9	22	4.1%	0.70 [-4.72, 6.12]
Qiu XS et al. 2020	47.99	11.09	34	31.08	10.48	51	4.4%	16.91 [12.20, 21.62]
Rose N et al. 2022	40.44	3.58	50	23.32	9.42	70	5.2%	17.12 [14.70, 19.54]
Turoni L et al. 2022	56	21	10	50.3	23	45	1.6%	5.70 [-8.95, 20.35]
Wu TL et al. 2019	40.9	8.2	43	32.1	9.8	5	2.9%	8.80 [-0.13, 17.73]
Xu QQ et al.(2021)(a)	19	12	15	25	10	19	3.4%	-6.00 [-13.56, 1.56]
Xu QQ et al.(2021)(b)	42	9	12	32	18	19	2.7%	10.00 [0.44, 19.56]
Zhang HY et al.(2022)(a)	47	4	19	33	10	33	4.7%	14.00 [10.14, 17.86]
Zhang HY et al.(2022)(b)	36.78	11.62	33	37.28	12.87	18	3.5%	-0.50 [-7.65, 6.65]
Zhang J et al. 2016	50	11	42	31	12	48	4.4%	19.00 [14.25, 23.75]
Zheng DQ et al. 2023	42.52	4.74	69	36.22	4.95	89	5.4%	6.30 [4.78, 7.82]
**Total (95% CI)**			**1022**			**1081**	**100.0%**	**10.47 [8.27, 12.67]**

Heterogeneity: Tau² = 22.81; Chi² = 176.87, df = 24 (P < 0.00001); I² = 86%
Test for overall effect: Z = 9.32 (P < 0.00001)

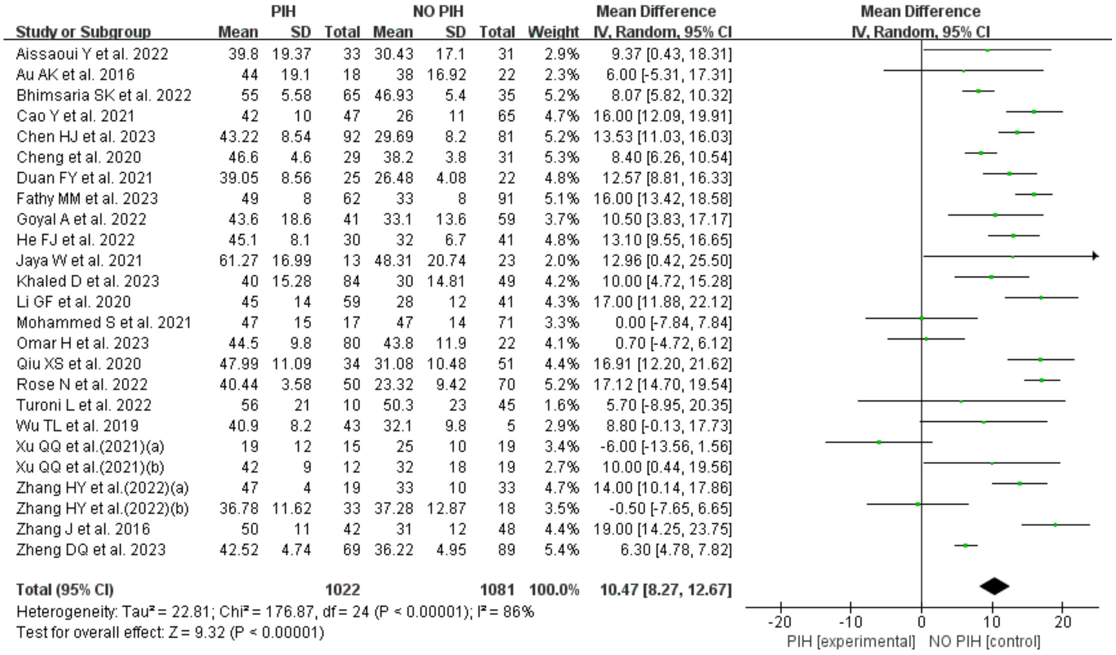

Figure S2B: Forest plot for the mean difference of DIVCmax between patients with PIH and without PIH.

Study or Subgroup	PIH Mean	PIH SD	PIH Total	NO PIH Mean	NO PIH SD	NO PIH Total	Weight	Mean Difference IV, Random, 95% CI
Au AK et al. 2016	1.6	0.302	18	1.7	0.451	22	3.7%	-0.10 [-0.33, 0.13]
Bhimsaria SK et al. 2022	1.04	0.23	65	1.5	0.77	35	3.4%	-0.46 [-0.72, -0.20]
Cao Y et al. 2021	1.53	0.22	47	1.45	0.28	65	5.7%	0.08 [-0.01, 0.17]
Chen HJ et al. 2023	1.88	0.2	92	1.94	0.22	81	6.0%	-0.06 [-0.12, 0.00]
Cheng et al. 2020	1.6	0.1	29	1.8	0.1	31	6.1%	-0.20 [-0.25, -0.15]
Duan FY et al. 2021	1.37	0.27	25	1.7	0.33	22	4.5%	-0.33 [-0.50, -0.16]
Goyal A et al. 2022	1.3	0.33	41	1.48	0.4	59	5.0%	-0.18 [-0.32, -0.04]
He FJ et al. 2022	1.7	0.13	30	1.76	0.17	41	5.9%	-0.06 [-0.13, 0.01]
Khaled D et al. 2023	1.55	0.36	84	1.6	0.44	49	5.0%	-0.05 [-0.20, 0.10]
Li GF et al. 2020	1.48	0.34	59	1.92	0.42	41	4.8%	-0.44 [-0.60, -0.28]
Mohammed S et al. 2021	1.48	0.29	17	1.43	0.36	71	4.7%	0.05 [-0.11, 0.21]
Qiu XS et al. 2020	1.42	0.49	34	1.72	0.49	51	4.0%	-0.30 [-0.51, -0.09]
Rose N et al. 2022	1.88	0.26	50	1.93	0.39	70	5.4%	-0.05 [-0.17, 0.07]
Turoni L et al. 2022	1	0.4	10	1.3	0.4	45	3.2%	-0.30 [-0.57, -0.03]
Wu TL et al. 2019	1.6	0.22	43	1.91	0.03	5	5.9%	-0.31 [-0.38, -0.24]
Xu QQ et al.(2021)(a)	1.32	0.27	15	1.67	0.25	19	4.5%	-0.35 [-0.53, -0.17]
Xu QQ et al.(2021)(b)	0.86	0.31	12	1.47	0.4	19	3.5%	-0.61 [-0.86, -0.36]
Zhang HY et al.(2022)(a)	1.33	0.34	19	1.68	0.33	33	4.3%	-0.35 [-0.54, -0.16]
Zhang HY et al.(2022)(b)	1.27	0.38	33	1.61	0.3	18	4.3%	-0.34 [-0.53, -0.15]
Zhang J et al. 2016	1.6	0.4	42	2	0.3	48	4.9%	-0.40 [-0.55, -0.25]
Zheng DQ et al. 2023	1.54	0.29	69	1.87	0.47	89	5.3%	-0.33 [-0.45, -0.21]
**Total (95% CI)**			**834**			**914**	**100.0%**	**-0.23 [-0.30, -0.16]**

Heterogeneity: Tau² = 0.02; Chi² = 140.16, df = 20 (P < 0.00001); I² = 86%
Test for overall effect: Z = 6.31 (P < 0.00001)

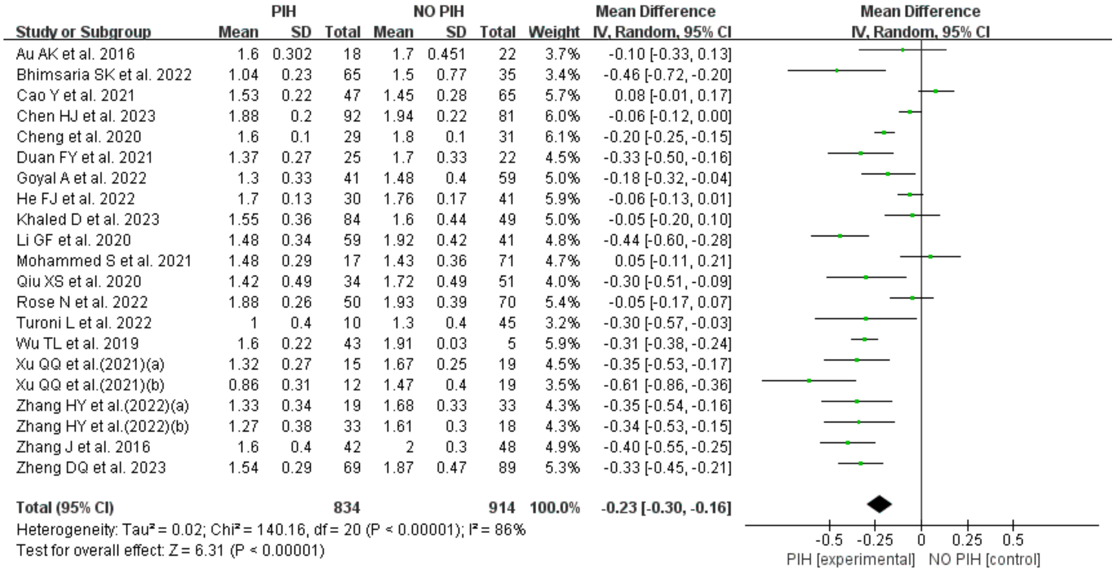

Figure S2C: Forest plot for the mean difference of DIVCmin between patients with PIH and without PIH.

	PIH			NO PIH				Mean Difference
Study or Subgroup	Mean	SD	Total	Mean	SD	Total	Weight	IV, Random, 95% CI
Au AK et al. 2016	0.9	0.402	18	1	0.338	22	7.0%	-0.10 [-0.33, 0.13]
Bhimsaria SK et al. 2022	0.65	0.38	65	0.56	0.23	35	8.0%	0.09 [-0.03, 0.21]
Duan FY et al. 2021	0.84	0.19	25	1.25	0.27	22	7.9%	-0.41 [-0.55, -0.27]
Goyal A et al. 2022	0.75	0.38	41	1.02	0.43	59	7.7%	-0.27 [-0.43, -0.11]
Khaled D et al. 2023	1.08	0.28	84	1.1	0.267	49	8.1%	-0.02 [-0.12, 0.08]
Mohammed S et al. 2021	0.81	0.36	17	0.77	0.32	71	7.5%	0.04 [-0.15, 0.23]
Rose N et al. 2022	1.11	0.17	50	1.48	0.35	70	8.1%	-0.37 [-0.46, -0.28]
Wu TL et al. 2019	0.93	0.17	43	1.31	0.21	5	7.4%	-0.38 [-0.57, -0.19]
Xu QQ et al.(2021)(a)	1.09	0.29	15	1.24	0.26	19	7.4%	-0.15 [-0.34, 0.04]
Xu QQ et al.(2021)(b)	0.5	0.2	12	1.03	0.45	19	7.0%	-0.53 [-0.76, -0.30]
Zhang HY et al.(2022)(a)	0.71	0.2	19	1.15	0.33	33	7.8%	-0.44 [-0.58, -0.30]
Zhang HY et al.(2022)(b)	0.81	0.29	33	1	0.13	18	8.0%	-0.19 [-0.31, -0.07]
Zheng DQ et al. 2023	0.78	0.35	69	1.63	0.44	89	8.0%	-0.85 [-0.97, -0.73]
**Total (95% CI)**			**491**			**511**	**100.0%**	**-0.28 [-0.43, -0.12]**

Heterogeneity: Tau² = 0.07; Chi² = 181.95, df = 12 (P < 0.00001); I² = 93%

Test for overall effect: Z = 3.57 (P = 0.0004)

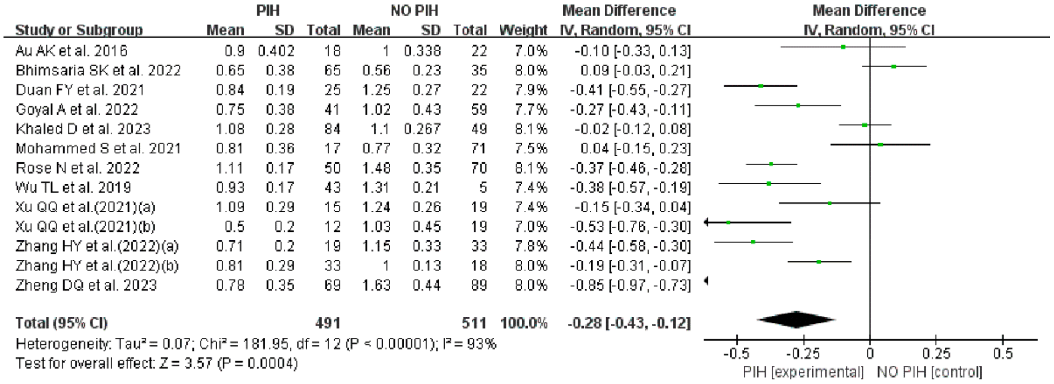

Figure S2D: Forest plot for the mean difference of carotid artery FTc between patients with PIH and without PIH.

	PIH			NO PIH				Mean Difference
Study or Subgroup	Mean	SD	Total	Mean	SD	Total	Weight	IV, Random, 95% CI
Chowdhury AR et al. 2023	316.3	22.67	29	352.8	46.67	31	14.3%	-36.50 [-54.88, -18.12]
Huang SS et al. 2023	323.21	15.11	73	346.2	21.11	39	22.0%	-22.99 [-30.47, -15.51]
Maitra et al. 2020	305.85	17.45	63	357.1	34.83	49	19.8%	-51.25 [-61.91, -40.59]
Wang J et al. 2022	355.5	22.9	63	386.3	19	36	21.4%	-30.80 [-39.20, -22.40]
Yang YY et al. 2023	320	15	44	340	20	56	22.4%	-20.00 [-26.86, -13.14]
**Total (95% CI)**			**272**			**211**	**100.0%**	**-31.52 [-42.19, -20.86]**

Heterogeneity: Tau² = 119.79; Chi² = 26.46, df = 4 (P < 0.0001); I² = 85%

Test for overall effect: Z = 5.79 (P < 0.00001)

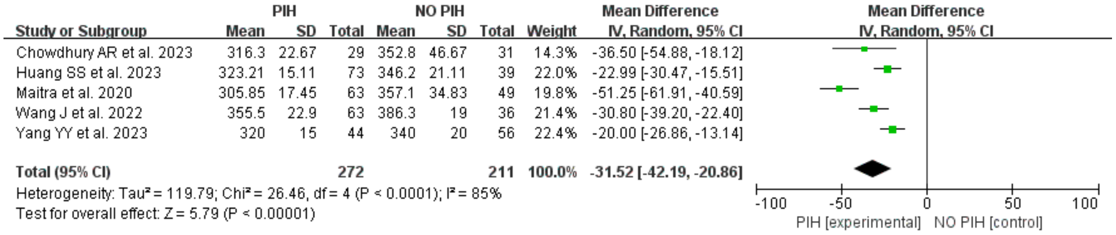

Figure S3A: Fagan's nomogram for IVC-CI.

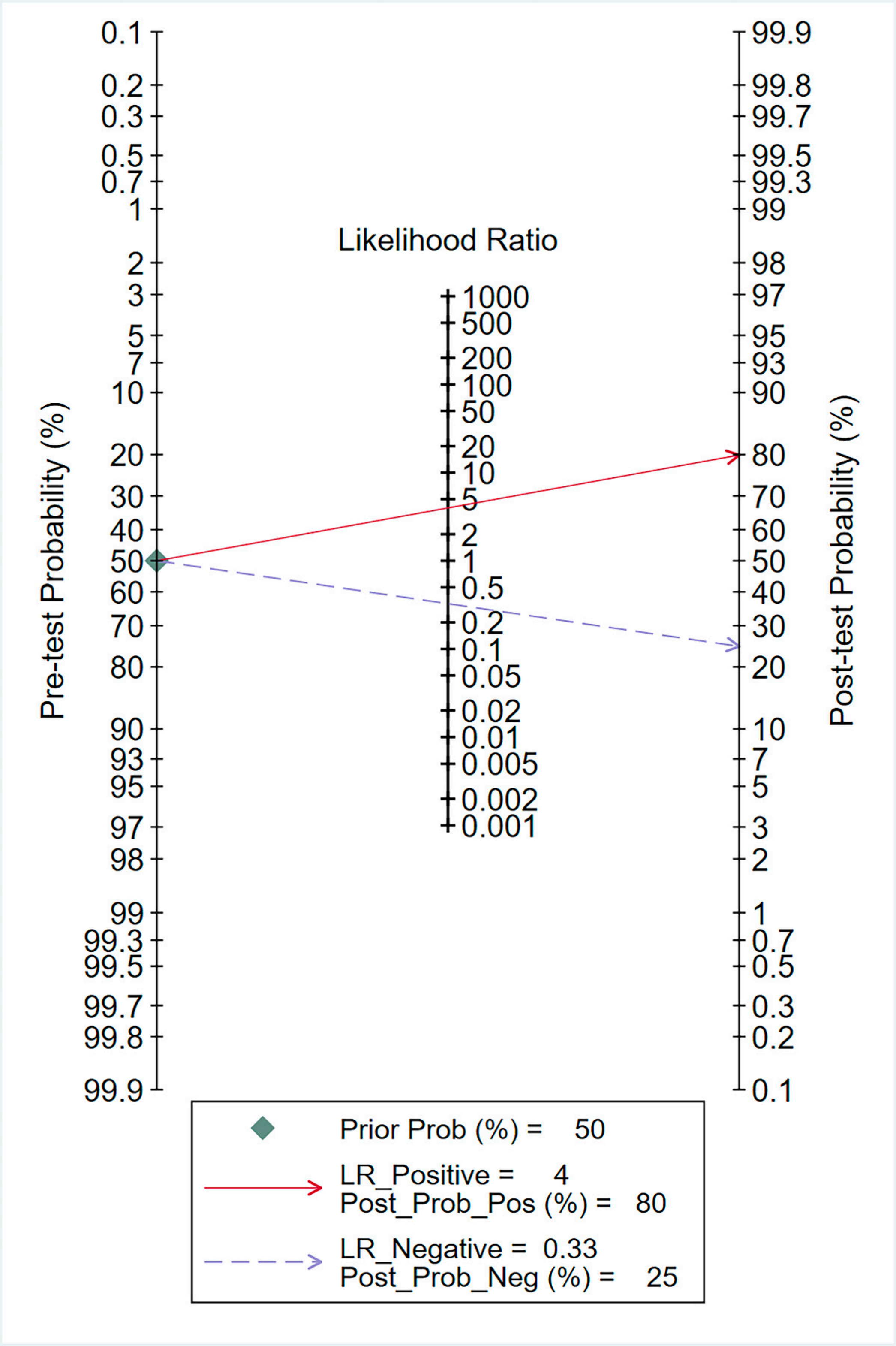

Figure S3B: Fagan's nomogram for DIVCmax.

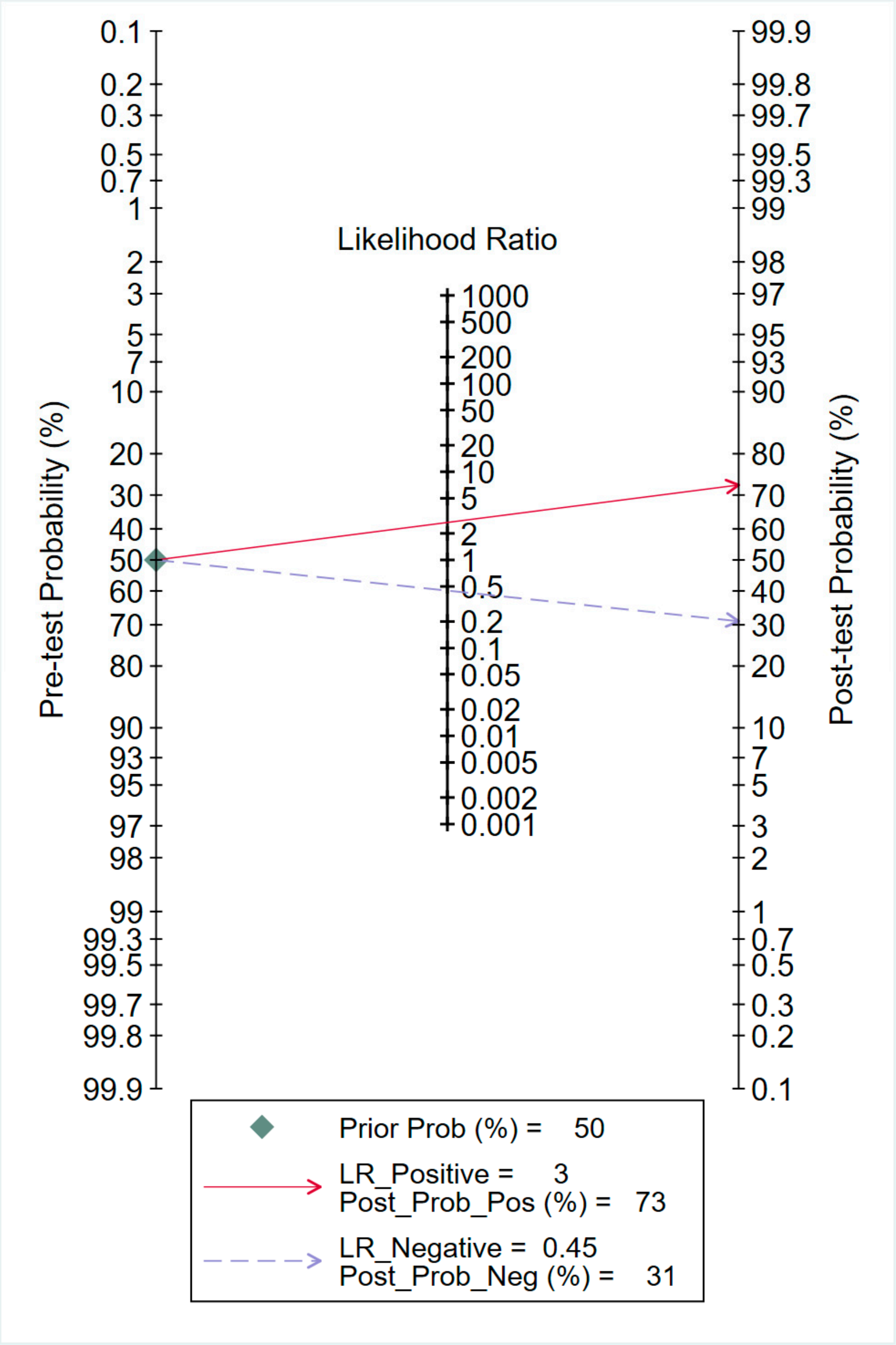

Figure S3C: Fagan's nomogram for DIVCmin.

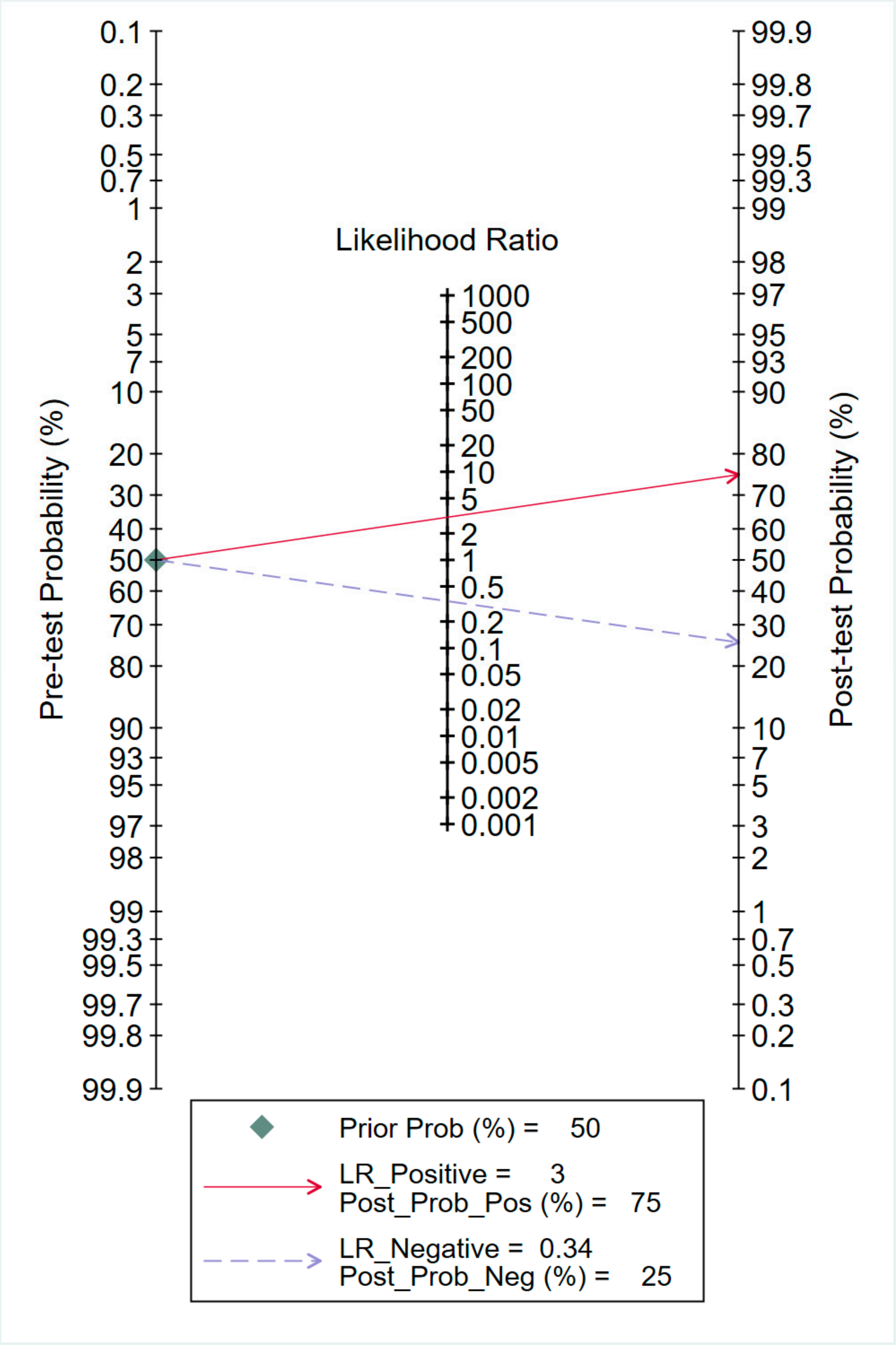

Figure S3D: Fagan's nomogram for carotid artery FTc.

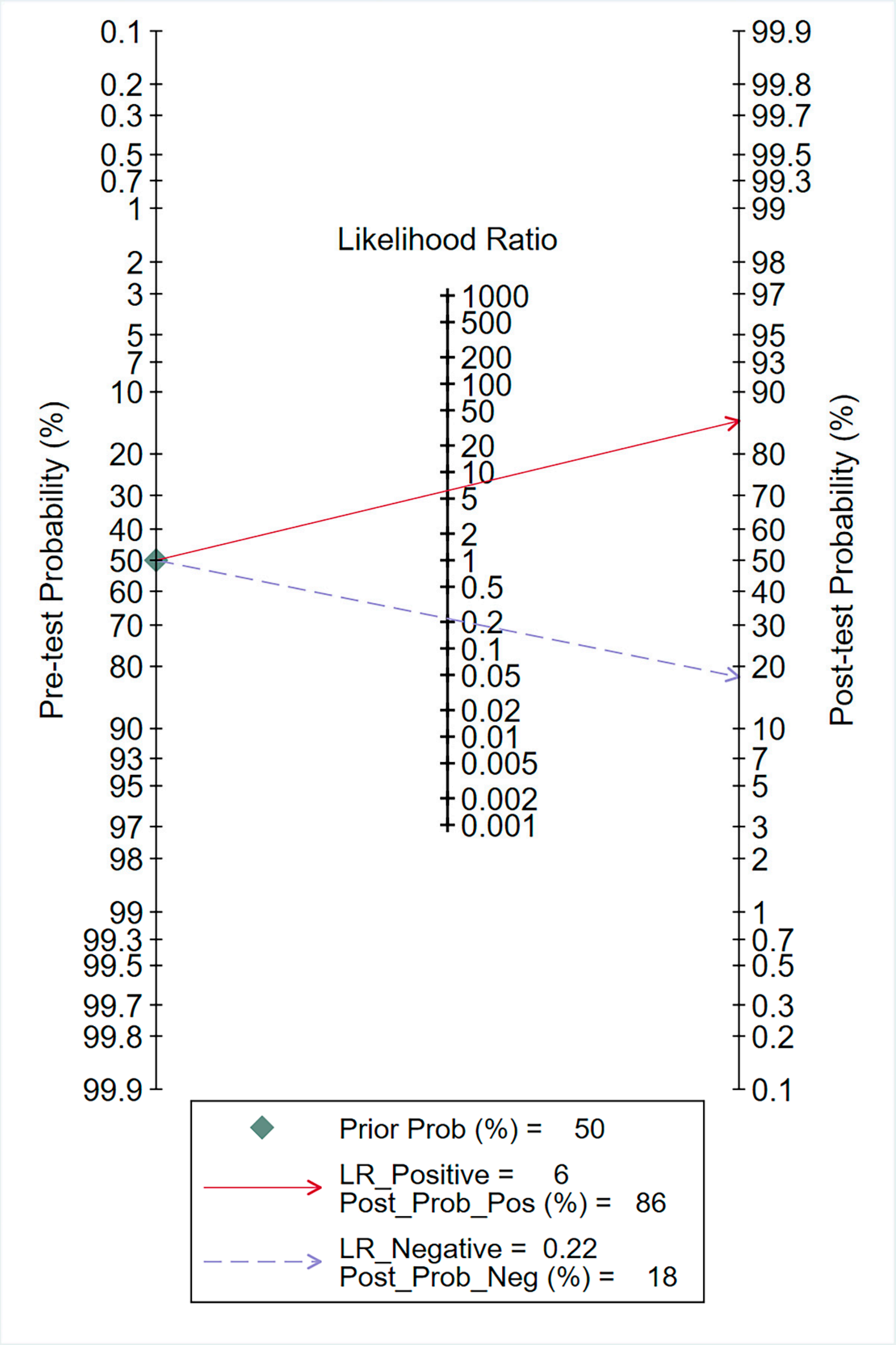

Figure S4A: Meta-regression for IVC-CI.

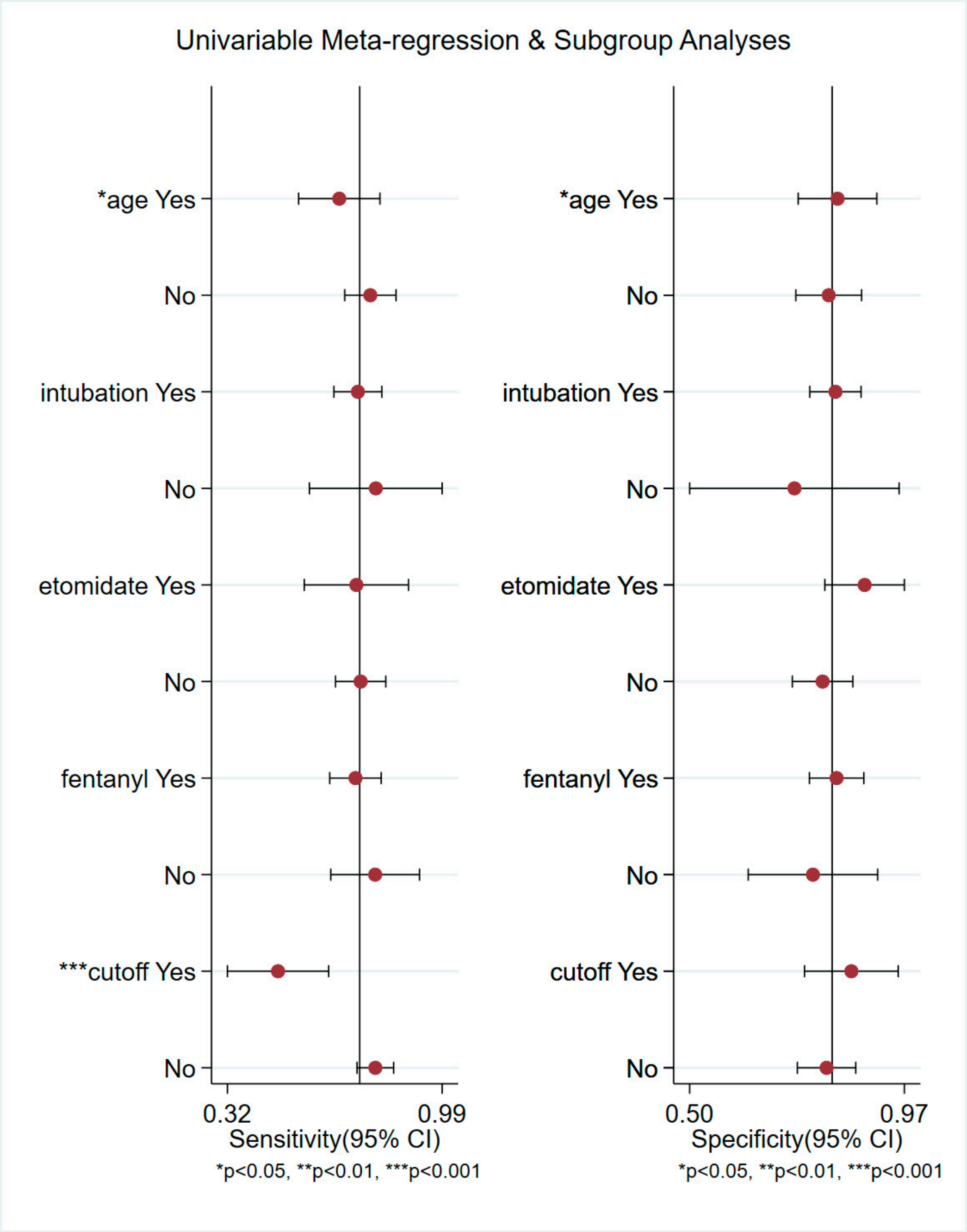

Notes: age: Yes = age > 60 years, No = other; etomidate: Yes = using etomidate for anesthesia induction, No = other; fentanyl: Yes = using fentanyl for anesthesia induction, No = other; cutoff: Yes = IVC-CI > 50%, No = other.

Figure S4B: Meta-regression for DIVCmax.

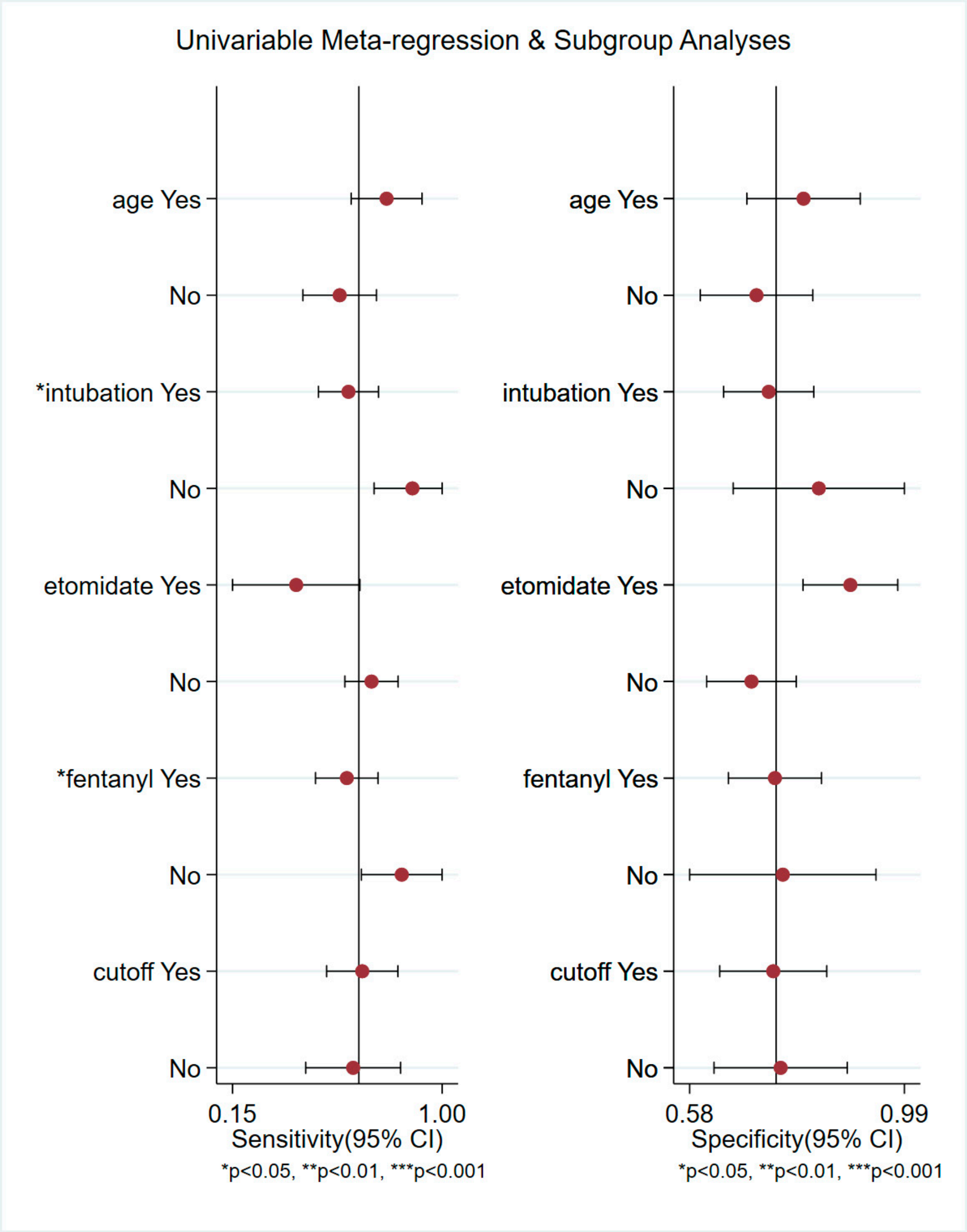

Notes: Yes = age > 60 years, No = other; etomidate: Yes = using etomidate for anesthesia induction, No = other; fentanyl: Yes = using fentanyl for anesthesia induction, No = other; cutoff: Yes = DIVCmax > 1.5cm, No = other.

Figure S4C: Meta-regression for DIVCmin.

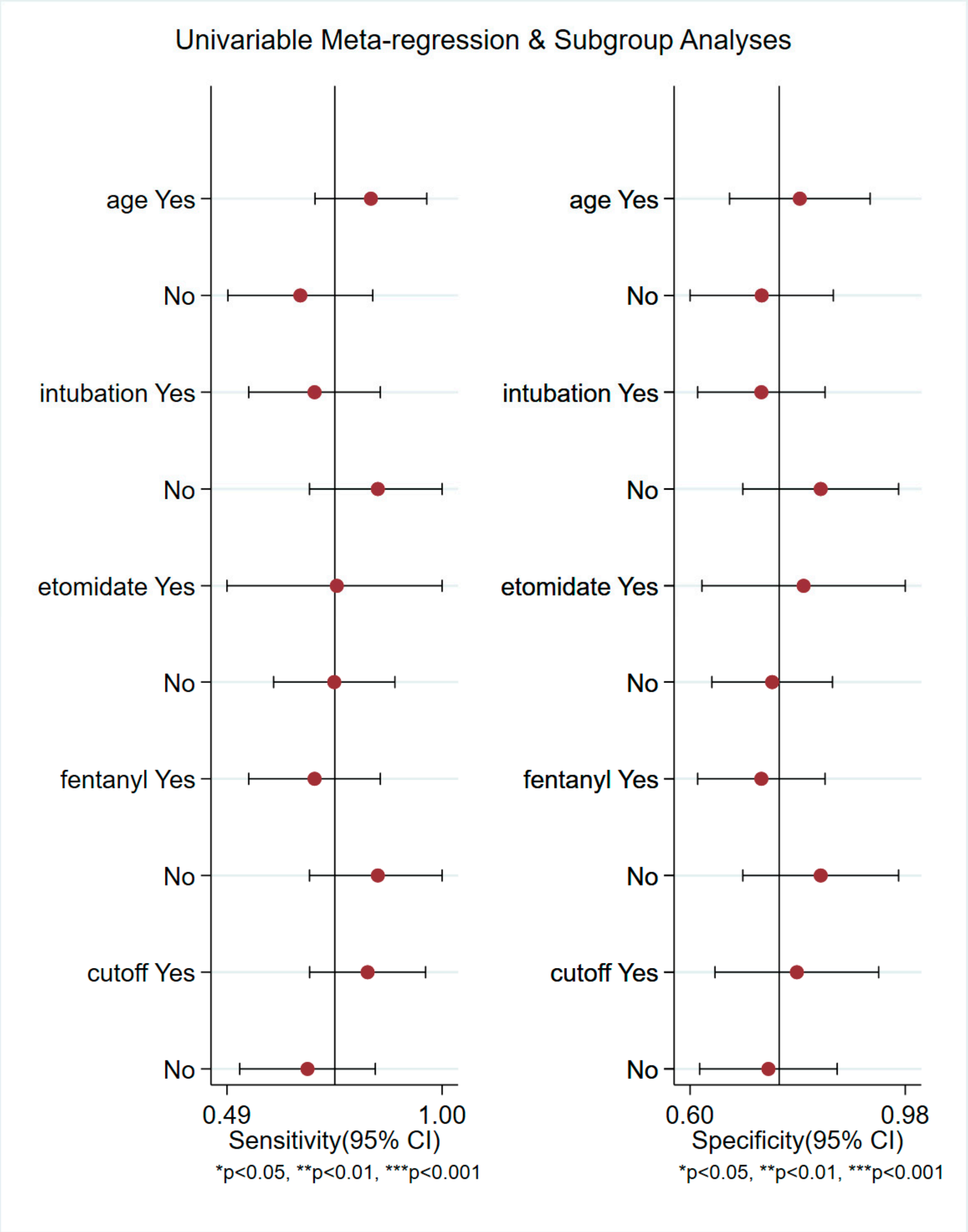

Notes: Yes = age > 60 years, No = other; etomidate: Yes = using etomidate for anesthesia induction, No = other; fentanyl: Yes = using fentanyl for anesthesia induction, No = other; cutoff: Yes = DIVCmin>0.9cm, No = other.

Figure S4D: Meta-regression for carotid artery FTc.

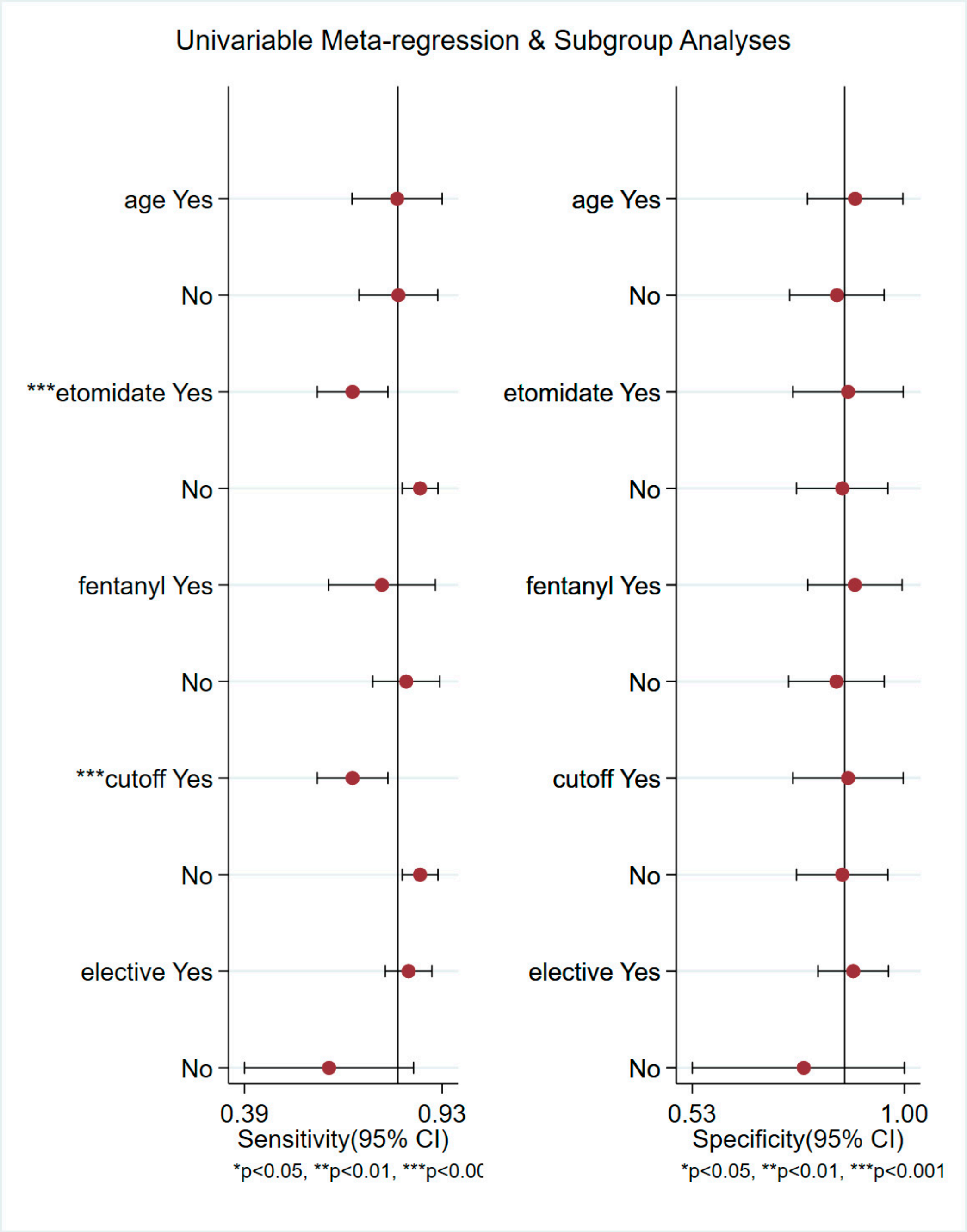

Notes: Yes = age > 60 years, No = other; etomidate: Yes = using etomidate for anesthesia induction, No = other; fentanyl: Yes = using fentanyl for anesthesia induction, No = other; cutoff: Yes = carotid artery FTc > 340ms, No = other.

Figure S5A: Deeks' funnel plot asymmetry test for publication bias of IVC-CI.

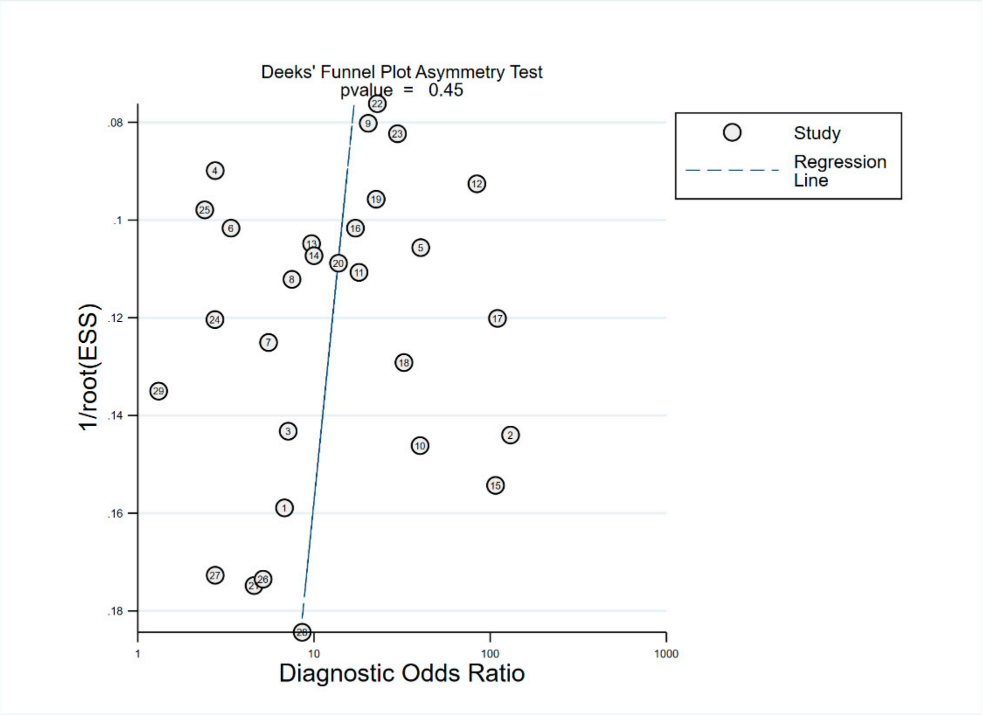

Figure S5B: Deeks' funnel plot asymmetry test for publication bias of DIVCmax.

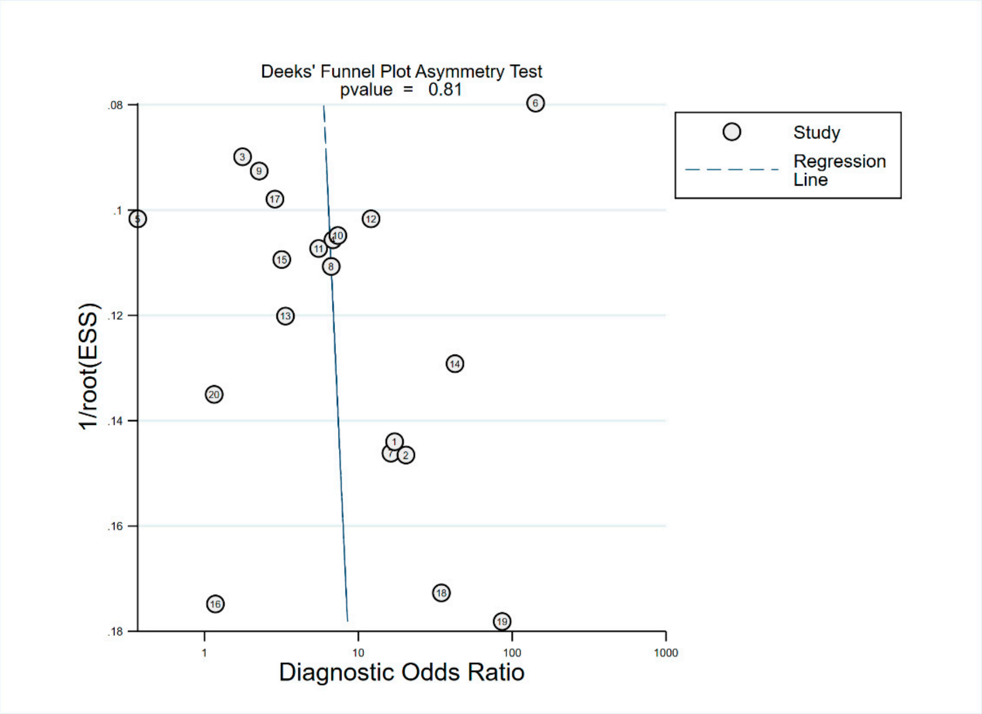

Figure S5C: Deeks' funnel plot asymmetry test for publication bias of DIVCmin.

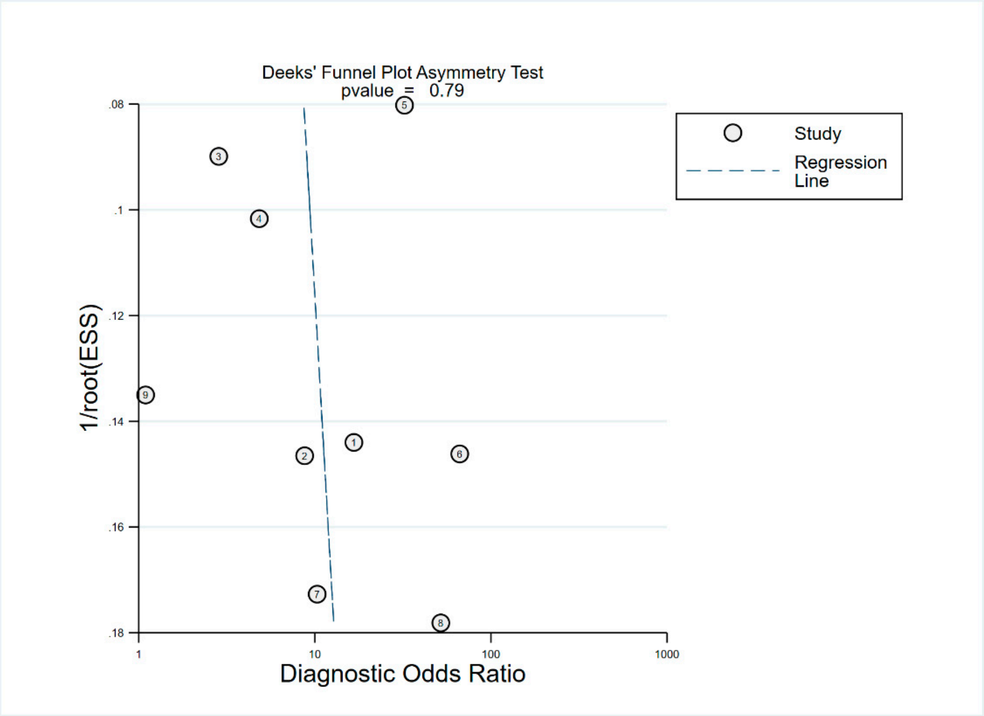

Figure S5D: Deeks' funnel plot asymmetry test for publication bias of carotid artery FTc.

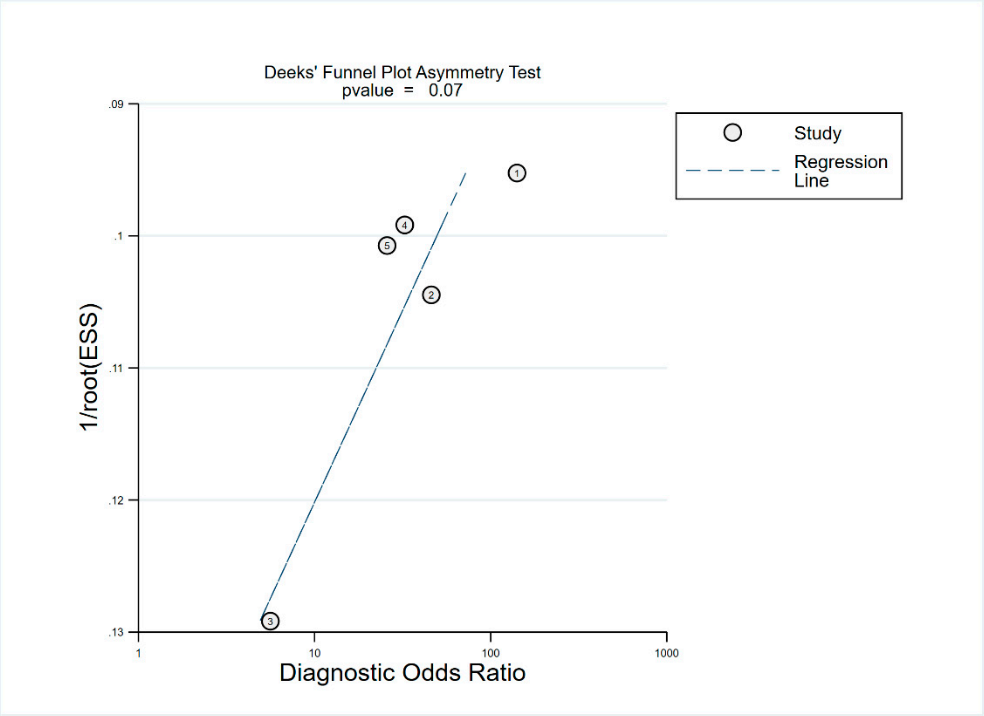

Figure S6: Assessment of Risk of Bias According to QUADAS-2.

	Risk of Bias				Applicability Concerns		
	Patient Selection	Index Test	Reference Standard	Flow and Timing	Patient Selection	Index Test	Reference Standard
Agarwal J et al. 2022	−	+	+	?	−	+	+
Aissaoui Y et al. 2022	+	+	+	+	+	+	+
Amin SR et al. 2022	+	+	+	?	+	+	+
Au AK et al. 2016	+	?	+	?	+	?	+
Bhimsaria SK et al. 2022	−	+	+	+	−	+	+
Cao Y et al. 2021	+	+	+	+	+	+	+
Cheng SS et al. 2020	+	+	+	+	+	+	+
Chen HJ et al. 2023	+	+	+	?	+	+	+
Chowdhury AR et al. 2023	−	?	?	+	?	?	+
Duan FY et al. 2021	+	?	+	+	+	+	+
Fathy MM et al. 2023	+	+	+	?	+	+	+
Goyal A et al. 2022	+	+	+	?	+	+	+
He FJ et al. 2022	+	+	+	?	+	+	+
Huang SS et al. 2023	+	?	?	+	+	+	−
Jaya W et al. 2021	+	?	?	+	+	+	+
Khaled D et al. 2023	?	+	+	?	?	+	+
Li GF et al. 2020	+	+	+	+	+	+	+
Maitra S et al. 2020	+	+	?	+	+	+	+
Mohammed S et al. 2021	+	?	−	?	+	?	+
Omar H et al. 2023	+	+	+	?	+	+	+
Purshothaman SS et al. 2020	?	+	+	?	?	+	+
Qiu XS et al. 2020	+	+	+	+	?	+	+
Rose N et al. 2022	+	+	?	+	+	+	+
Sari S et al. 2019	+	+	+	+	+	+	+
Szabo M et al. 2019	?	+	+	?	?	−	+
Turoni L et al. 2022	+	+	+	+	?	+	+
Wang J et al. 2022	+	?	+	+	+	+	+
Xu QQ et al. 2021	+	+	+	?	+	+	+
Yang YY et al. 2023	?	?	?	+	+	+	+
Zhang HY et al. 2022	?	+	+	?	+	+	+
Zhang J et al. 2016	+	+	+	?	+	+	?
Zheng DQ et al. 2023	+	+	+	+	+	+	+

− **High** ? **Unclear** + **Low**
